# Activatable Sulfur Dioxide Nanosonosensitizer Enables Precisely Controllable Sono‐Gaseous Checkpoint Trimodal Therapy for Orthotopic Hepatocellular Carcinoma

**DOI:** 10.1002/advs.202409442

**Published:** 2024-12-16

**Authors:** Jing Liang, Guangwen Cheng, Luping Qiu, Liyun Xue, Huning Xu, Xiaohui Qiao, Na Guo, Huijing Xiang, Yu Chen, Hong Ding

**Affiliations:** ^1^ Department of Ultrasound Huashan Hospital Fudan University Shanghai 200040 China; ^2^ Department of Pathology Zhejiang Cancer Hospital Hangzhou Zhejiang 310022 China; ^3^ Materdicine Lab School of Life Sciences Shanghai University Shanghai 2000444 China

**Keywords:** gas therapy, immunotherapy, nanomedicine, nanosonosensitizer, sonodynamic therapy

## Abstract

Immune checkpoint blockade (ICB) is combined with sonodynamic therapy (SDT) to increase response rates and enhance anticancer efficacy. However, the “always on” property of most sonosensitizers in reducing tumor microenvironment (TME) compromises the therapeutic outcome of sonoimmunotherapy and exacerbates adverse side effects. Precisely controllable strategies combining sulfur dioxide (SO_2_) gas therapy with cancer immunotherapy can address these issues but remain lacking. Herein an “activatable SO_2_ nanosonosensitizer” for precise sono‐gaseous checkpoint trimodal therapy of orthotopic hepatocellular carcinoma (HCC) is reported, whose full activity is initiated by ultrasound (US) irradiation in the reducing TME. This “activatable SO_2_ nanosonosensitizer,” Aza‐DNBS nanoparticles (NPs), are established by self‐assembling Aza‐boron‐dipyrromethene based sonosensitizer molecules and 2,4‐dinitrobenzenesulfonate (DNBS)‐caged SO_2_ prodrug. The activity of Aza‐DNBS NPs is initially silenced, and the sonodynamic, gaseous, and immunosuppressive TME reprogramming activities are precisely awakened under US irradiation. Due to the glutathione‐responsiveness of Aza‐DNBS NPs, Aza‐DNBS NPs can generate large amounts of SO_2_ for gas therapy‐enhanced SDT, which triggers robust immunogenic cell death activation and reprogramming of the immunosuppressive TME, thereby significantly suppressing orthotopic tumor growth and delaying lung metastasis. Thus, this study represents a strategy for designing a generic nanoplatform for precisely combined immunotherapy of orthotopic HCC.

## Graphical abstract

An activatable SO_2_ nanosonosensitizer is designed, denoted as Aza‐DNBS NPs for precise sono‐gaseous checkpoint trimodal therapy of orthotopic HCC. Under US irradiation, Aza‐DNBS NPs can efficiently generate ROS to induce SDT. Additionally, Aza‐DNBS NPs facilitated the rapid and controllable release of SO_2_ in the GSH‐overexpressing TME for gas therapy‐enhanced SDT, enabling robust ICD activation and reprogramming of the immunosuppressive TME.

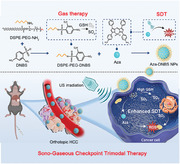

## 1 Introduction

Hepatocellular carcinoma (HCC) remains a significant global health concern due to its high mortality rate and poor prognosis.^[^ ^1^ ^]^ Immune checkpoint blockade (ICB) has shown the potential to revolutionize cancer treatment, including HCC.^[^ ^2^ ^]^ Nevertheless, only a small fraction of patients respond positively to this approach. This limited response may be attributed in part to inadequate tumor immunogenicity and the immunosuppressive tumor microenvironment (TME).^[^ ^3^ ^]^ Numerous strategies are currently being explored to enhance ICB therapy in HCC, focusing primarily on combining ICB with other therapies.^[^ ^4^ ^]^ A growing body of evidence from numerous studies has suggested that sonodynamic therapy (SDT) is effective in synergistically treating tumors with immune checkpoint blockade (ICB).^[^ ^5^ ^]^ The capacity of sonosensitizers to selectively generate toxic reactive oxygen species (ROS) in tumor regions remains one of the crucial determinants of maximizing the treatment efficacy of sono‐immunotherapy while minimizing side effects on healthy tissues. However, most sonosensitizers are in the “always on” phase, and singlet oxygen (^1^O_2_) can be produced once the sonosensitizers are exposed to ultrasound (US) irradiation even in normal cells, resulting in undesirable side effects.^[^ ^6^ ^]^ Activatable sonosensitizers that conjugated with caged groups are initially inactive and their activity can be initiated by a specific stimulus.^[^ ^7^ ^]^ Therefore, engineering activatable sonosensitizers is highly important for precise sono‐immunotherapy with minimal adverse effects on adjacent healthy tissues.

While the ROS produced by activatable sonosensitizers can effectively induce cell death, tumor cells can respond to the SDT‐induced oxidative stress by upregulating glutathione (GSH) expression, thereby reducing the therapeutic effect of SDT.^[^ ^8^ ^]^ Currently, the possible mechanism of sulfur dioxide (SO_2_) gas therapy could address these issues, involving the depletion of overexpressed GSH and the modulation of oxidative stress by elevating ROS levels. This therapy reveals significant therapeutic potential in a range of health disorders.^[^ ^9^ ^]^ Especially in tumor treatment, the SO_2_ produced within cells is in a gaseous form. This unique characteristic allows it to overcome the hurdle of tumor heterogeneity, allowing it to diffuse freely into deep tumor tissues and thereby inducing the death of tumor cells located deep within the tissues.^[^ ^10^ ^]^ In addition, SO_2_ enhances ROS production,^[^ ^11^ ^]^ possibly bolstering immunogenic cell death (ICD) of tumor cells and subsequent ICD‐induced immune responses. Hence, the incorporation of an SO_2_ prodrug may synergistically boost the effectiveness of sono‐immunotherapy, thereby amplifying the antitumor immune responses. However, high doses of SO_2_ can cause damage to normal tissues. Precise release of SO_2_ in time, space, and dose is essential for on‐demand gas therapy.^[^ ^12^ ^]^ Several previous studies have validated that the controlled release of SO_2_ is highly promising for tumor therapy.^[^ ^13^ ^]^ However, the poor biodegradability and biocompatibility of current inorganic SO_2_ donors limit their potential use. Moreover, the construction of “activatable sonosensitizers” for SO_2_ gas therapy‐augmented sono‐immunotherapy is still lacking. Therefore, there is an urgent need to develop multifaceted “activatable sonosensitizers” with high biocompatibility and biodegradability for sono‐immunotherapy and on‐demand release of SO_2_ in tumor tissues.

**Scheme 1:**
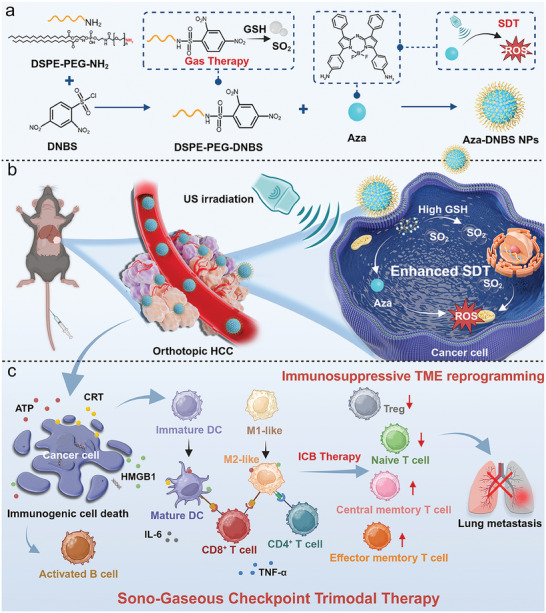
Schematic illustration of Aza‐DNBS NPs‐mediated sono‐gaseous checkpoint trimodal therapy for orthotopic HCC.

Herein, we designed an activatable SO_2_ nanosonosensitizer, denoted as Aza‐DNBS nanoparticles (NPs) for precise sono‐gaseous checkpoint trimodal therapy of HCC through self‐assembly of aza‐boron‐dipyrromethene‐based sonosensitizer molecules and a 2,4‐dinitrobenzenesulfonate (DNBS)‐caged SO_2_ prodrug. Under US irradiation, Aza‐DNBS NPs can efficiently generate a large amount of ROS to induce SDT. Additionally, Aza‐DNBS NPs facilitate the rapid and controllable release of SO_2_ in the GSH‐overexpressing TME for gas therapy‐enhanced SDT, enabling robust ICD activation and reprogramming of the immunosuppressive TME. In vivo, assessments using orthotopic mouse HCC and HCC lung metastasis models confirmed that synergistic gas therapy and SDT effects in combination with anti‐programmed death ligand 1 antibody (αPD‐L1) markedly inhibited orthotopic tumor proliferation and lung metastasis. Transcriptome sequencing analysis illustrated that Aza‐DNBS NPs combined with the US promoted the reprogramming of the immunosuppressive TME, facilitated the activation of T cells and B cells, and decreased the levels of immunosuppressive modulators, thereby enhancing ICB efficacy and systemic memory immunity (**Scheme** **1**). Therefore, this work represents an innovative strategy for designing a universal nanoplatform to reverse the immunosuppressive TME in the treatment of orthotopic HCC.

## 2 Results and Discussion

### 2.1 Fabrication and Characterization of Aza‐DNBS NPs

**Figure 1:**
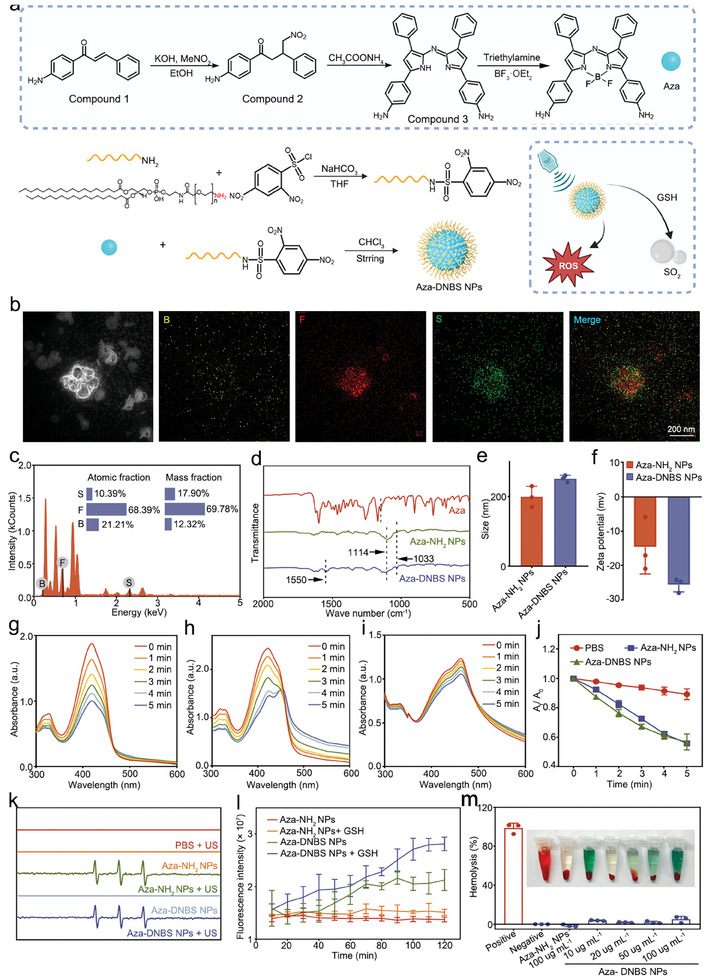
Synthesis and Characterization of the Aza‐DNBS NPs. a) Schematic diagram of the synthesis process of the Aza‐DNBS NPs. b,c) HAADF STEM image and corresponding element maps showing the distributions of B, F, and S in the Aza‐DNBS NPs. d) FTIR spectra of Aza, Aza‐NH_2_ NPs, and Aza‐DNBS NPs. e) Hydrodynamic sizes and f) zeta potentials of Aza‐DNBS NPs and Aza‐NH_2_ NPs (*n* = 3). g–i) UV/Vis absorption spectra of DPBF aqueous solutions containing g) Aza‐NH_2_ NPs, h) Aza‐DNBS NPs, and i) PBS under US irradiation. j) Relative changes in the absorbance of DPBF solution at 415 nm after various treatments. k) ESR spectra of various treatment groups using TEMP as a ^1^O_2_ capturing agent. l) Identification of the SO_2_ derivative HSO_3_^−^ in NPs solutions with or without GSH using a DEACA probe (*n* = 3). m) Hemolysis assay of Aza‐NH_2_ NPs and Aza‐DNBS NPs (*n* = 3).

As shown in **Figure** **1**a, to facilitate efficient SDT, aza‐boron‐dipyrromethene (Aza) was chosen as the sonosensitizer due to its ability to generate large amounts of ROS when exposed to US irradiation.^[^ ^14^ ^]^ UV/Vis spectroscopy was performed to monitor the intermediates during the synthesis process (Figure S1, Supporting Information). The chemical structure of Aza was verified by ^1^H NMR (Figure S2, Supporting Information). To ensure biocompatibility and GSH‐responsive SO_2_ release, Aza was modified with 1,2‐distearoyl‐sn‐glycero‐3‐phosphoethanolamine‐N‐[meth‐oxy(polyethylene glycol)‐2000] (DSPE‐PEG‐NH_2_), incorporating with SO_2_ prodrug 2,4‐dinitrobenzenesulfonyl chloride (DNBS). This modification led to the formation of Aza‐DNBS NPs. For comparison, the non‐SO_2_ prodrug Aza‐NH_2_ NPs were synthesized by self‐assembling Aza and DSPE‐PEG‐NH_2_. High‐magnification high‐angle annular dark‐field (HAADF) images, along with corresponding energy dispersive spectroscopy (EDS) mapping images of Aza‐DNBS NPs revealed the formation of irregular particles with a size of ≈200 nm (Figure 1b). Boron (B), fluorine (F), and sulfur (S) were all located in Aza‐DNBS NPs. B and F elements were primarily distributed in the interior of Aza‐DNBS NPs, and S element was mainly distributed on the surface. The contents of B, F, and S elements were 12.32, 69.78, and 17.90 wt.%, respectively (Figure 1c). Furthermore, the Fourier transform infrared (FTIR) spectrum of the Aza‐DNBS NPs displayed the characteristic peaks at 1550, 1114, and 1033 cm^−1^, attributed to the vibrations of the AR‐NO_2_ bonds in DNBS, the C‐H bonds in Aza, and the P = O bonds in DSPE‐PEG‐NH_2_, respectively (Figure 1d). These findings further indicate the successful fabrication of the Aza‐DNBS NPs. Dynamic light scattering (DLS) measurements indicated that the hydrodynamic sizes of the Aza‐DNBS NPs and Aza‐NH_2_ NPs were 247.67 ± 6.04 nm and 198.93 ± 14.21 nm, respectively. The addition of the SO_2_ prodrug to the NPs surface slightly increased the particle size. Additionally, the zeta potential decreased from −14.62 ± 7.82 mV for the Aza‐NH_2_ NPs to −25.61 ± 2.04 mV for the Aza‐DNBS NPs (Figure 1e,f). The change in zeta potential also verified the effective construction of the Aza‐DNBS NPs. Furthermore, to assess the stability of Aza‐DNBS NPs, we dissolved them in phosphate‐buffered saline (PBS) and PBS supplemented with 10% fetal bovine serum (FBS) and observed changes in polydispersity index over 7 days. The results demonstrated that Aza‐DNBS NPs exhibited good stability in both PBS and 10% FBS solution (Figure S3, Supporting Information).

To monitor the generation of ^1^O_2_ by Aza‐NH_2_ NPs and Aza‐DNBS NPs under US irradiation (1.0 MHz, 1 W cm^−2^), Aza‐NH_2_ NPs and Aza‐DNBS NPs were dissolved in PBS, and a probe called 1,3‐diphenylbenzofuran (DPBF) was used. The UV/Vis spectra showed that the absorbance of DPBF quickly decreased at 415 nm in the Aza‐NH_2_ NPs + US and Aza‐DNBS NPs + US groups, with the A_5_ _min_/A_0_ _min_ values of 0.57 ± 0.05 and 0.56 ± 0.01, respectively. In contrast, the decrease in the absorbance of DPBF at 415 nm was negligible when PBS was exposed to US radiation (Figure 1g–j). Additionally, electron spin resonance (ESR) spectroscopy, employing the trapping agent, 2,2,6,6‐tetramethylpiperidine (TEMP), was conducted to confirm the generation of ^1^O_2_. A distinctive three‐line ESR signal with a relative intensity of 1:1:1 was noticed in the Aza‐NH_2_ NPs and Aza‐DNBS NPs under US irradiation, indicating the presence of the ^1^O_2_/TEMP adduct (Figure 1k; Figure S4, Supporting Information). To evaluate the GSH‐responsive SO_2_ release capacity of the Aza‐DNBS NPs, fluorescence spectroscopy was performed using a fluorescent probe called 7‐diethylaminocoumarin‐3‐aldehyde (DEACA). Once released and dissolved in water, SO_2_ exists in the form of bisulfite (HSO_3_^−^), which can be detected and quantified by DEACA without interference from other ions.^[^ ^15^ ^]^ As shown in Figure 1l, the fluorescence intensity of the mixture containing Aza‐DNBS NPs and GSH gradually increased over time. However, the fluorescence intensity of Aza‐NH_2_ NPs with or without GSH remained almost unchanged. These results confirmed that Aza‐DNBS NPs could not only produce ^1^O_2_ for efficient SDT, but also release SO_2_ in response to GSH. On the other hand, Aza‐NH_2_ NPs could only generate ^1^O_2_ but did not possess the ability to release SO_2_. To ascertain the biosafety of intravenous administration of these NPs, we measured their hemolytic activity. Both the Aza‐NH_2_ NPs and Aza‐DNBS NPs exhibited marginal hemolytic activity (percent hemolysis < 5%), at a concentration of 100 µg mL^−1^ (Figure 1m).

### 2.2 Tumor Cell Apoptosis Induced by Aza‐DNBS NPs Under US Irradiation

**Figure 2:**
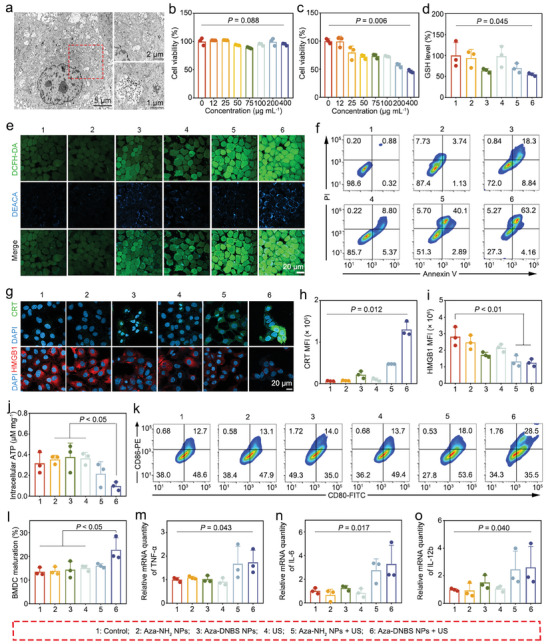
Therapeutic efficacy of Aza‐DNBS NPs under US irradiation in vitro. a) Representative bio‐TEM images depicting the intracellular uptake of the Aza‐DNBS NPs. b,c) Viability rates of b) L‐02 cells and c) Huh7 cells after treatment with various concentrations of Aza‐DNBS NPs (*n* = 3). The data were analyzed by one‐way ANOVA. d) Intracellular GSH levels in Huh7 cells under different treatments (*n* = 3), with statistical significance determined by the Kruskal‐Wallis test. e) CLSM images of Huh7 cells labeled with DCFH‐DA and DEACA after various treatments. f) Flow cytometry analysis of the cytotoxic effects of Aza‐DNBS NPs on H22 cells. g) Immunofluorescence analysis of the expression of CRT and HMGB1 in Huh7 cells after different treatments, and the corresponding mean fluorescence intensity (MFI) of (h) CRT and i) HMGB1 (*n* = 3). Statistical significance was calculated through the Kruskal‐Wallis test. j) Intracellular ATP expression levels in Huh7 cells subjected to different treatments (*n* = 3), analyzed by one‐way ANOVA. k) Flow cytometry analysis of BMDCs maturation (CD80^+^CD86^+^ proportion in CD11c^+^ cells, *n* = 3) and l) the corresponding statistical analysis results. Statistical significance was calculated through the Kruskal‐Wallis test. m–o) Relative gene expression levels of m) *TNF‐α*, n) *IL‐6*, and o) *IL‐12b* in BMDCs after different treatments (*n* = 3). The expression levels of these genes were normalized to that of *β‐actin*. Statistical significance was determined through the Kruskal–Wallis test.

Next, we explored the ability of Aza‐DNBS NPs to facilitate cellular uptake and maintain antitumor performance. Experiments were conducted using the human hepatocellular carcinoma cell line Huh7, which was treated with Aza‐DNBS NPs for 4 h. Subsequent bio‐TEM images revealed successful uptake of the Aza‐DNBS NPs by the cells, with dispersion observed in the cytoplasm (**Figure** **2**a; Figure S5, Supporting Information). To further evaluate the cytotoxicity of Aza‐DNBS NPs, a normal human hepatocyte line (L‐02) and Huh7 cells were incubated with varying doses of Aza‐DNBS NPs for 24 h. The results showed that the cell viability of L‐02 cells was virtually unaffected, even at high concentrations of Aza‐DNBS NPs (up to 400 µg mL^−1^), suggesting the high biocompatibility of Aza‐DNBS NPs with normal cells (Figure 2b). On the other hand, an observable decrease in Huh7 cell viability was correlated with an increase in the concentration of the Aza‐DNBS NPs. Specifically, the survival rate of Huh7 cells decreased to 45.04% at a concentration of 400 µg mL^−1^, suggesting that the reduction in the cell viability could be attributed to the concentration‐dependent SO_2_ release in the GSH‐overexpressing tumor environment, resulting in tumor cell mortality (Figure 2c).

Subsequently, we aimed to determine whether Aza‐DNBS NPs contribute to GSH depletion and to assess their efficacy in mediating SDT. As depicted in Figure 2d, intracellular GSH levels were significantly downregulated in Huh7 cells treated with 100 µg mL^−1^ Aza‐DNBS NPs regardless of US irradiation exposure. Concurrent with the decrease in intracellular GSH, confocal laser scanning microscopy (CLSM) confirmed the generation of intracellular SO_2_. We also observed the release of SO_2_ from Huh7 cells at different time points after coincubation with the Aza‐DNBS NPs. As shown in Figure S6 (Supporting Information), the SO_2_ signal gradually increased over 12 h, remained slightly detectable at 24 h, and was almost undetectable at 48 h. In addition, Aza‐DNBS NPs generated substantial amounts of ROS after exposure to US irradiation (1.0 MHz, 1 W cm^−2^, 50% duty cycle, 3 min), as detected by the ROS fluorescent indicator, 2′,7′‐dichlorodihydrofluorescein diacetate (DCFH‐DA). In contrast, Aza‐NH_2_ NPs could only produce ROS when the cells were exposed to US irradiation (Figure 2e). Notably, Aza‐DNBS NPs alone were capable of producing a small amount of ROS, and at identical concentrations, Aza‐DNBS NPs + US generated more ROS than did Aza‐NH_2_ NPs + US (Figure S7, Supporting Information). This suggested that the released SO_2_ promoted the production of intracellular ROS. Moreover, the in vitro anticancer effect of Aza‐DNBS NPs under US irradiation was evaluated using calcein acetoxymethyl ester/propidium iodide (calcein‐AM/PI) and cell counting kit‐8 (CCK‐8) assays (Figure S8, Supporting Information). The results showed that Aza‐DNBS NPs alone manifested a certain degree of cytotoxicity to Huh7 cells. Under US irradiation, compared to Aza‐NH_2_ NPs, Aza‐DNBS NPs exhibited superior antitumor effects, suggesting that the released SO_2_ could synergize with SDT to destroy tumor cells more effectively. Flow cytometry analysis of the cytotoxic effects of Aza‐DNBS NPs on the mouse hepatocyte line, H22 cells, revealed similar results. Treatment with 100 µg mL^−1^ Aza‐DNBS NPs exhibited a modest anticancer effect with an apoptosis rate of 18.3%. However, after exposure to US irradiation, Aza‐DNBS NPs at the same concentration exerted the desired therapeutic efficacy (Figure 2f). These findings further indicate that the NPs‐mediated SDT significantly induced the apoptosis of cancer cells.

### 2.3 Enhanced ICD of Tumor Cells Induced by Aza‐DNBS NPs Under US Irradiation

Given the significant tumor cell apoptosis mediated by Aza‐DNBS NPs, we hypothesized that this effect may trigger the release of abundant damage‐associated molecular patterns (DAMPs) into the TME, potentially fostering immune stimulation mediated by derived dendritic cells (DCs). Immunofluorescence analysis revealed a marked increase in calreticulin (CRT) expression in Huh7 cells compared with that in the control group, while high mobility group box 1 (HMGB1) expression decreased rapidly following treatment with Aza‐DNBS NPs + US. A similar decrease in intracellular HMGB1 was also observed in the Aza‐NH_2_ NPs + US group (Figure 2g–i). Furthermore, flow cytometry analysis revealed a significant increase in CRT expression on dead cell membranes in the Aza‐DNBS NPs + US group (Figure S9, Supporting Information), accompanied by an increase in extracellular HMGB1 level (Figure S10a, Supporting Information). Additionally, we evaluated intracellular adenosine 5′‐triphosphate (ATP) expression and found a significant decrease in ATP levels in Huh7 cells treated with Aza‐DNBS NPs + US. A similar downward trend was observed in the Aza‐NH_2_ NPs + US group (Figure 2j). In contrast, the extracellular ATP level in the cell culture medium was significantly elevated in the Aza‐DNBS NPs + US group (Figure S10b, Supporting Information). These changes in CRT, HMGB1, and ATP levels after treatment with Aza‐DNBS NPs + US supported our hypothesis that treatment of Huh7 tumor cells with Aza‐DNBS NPs + US could enhance their immunogenicity by releasing abundant DAMPs, potentially facilitating subsequent DC maturation.

Consequently, we cocultured bone marrow‐derived dendritic cells (BMDCs) with H22 cells pretreated with various protocols for 24 h. Flow cytometry analysis was used to determine the proportion of CD80^+^CD86^+^ cells among CD11c^+^ cells to reflect the maturation of BMDCs. Evidently, both Aza‐NH_2_ NPs + US and Aza‐DNBS NPs + US promoted BMDCs maturation, resulting in increased numbers of CD80^+^CD86^+^ BMDCs (Figure 2k,l). Additionally, the relative expression levels of *tumor necrosis factor‐alpha* (*TNF‐a*), *interleukin 6* (*IL‐6*), and *IL‐12b* in BMDCs were significantly increased in the Aza‐NH_2_ NPs + US and Aza‐DNBS NPs + US groups (Figure 2m–o). Interestingly, the proportion of CD80^+^CD86^+^ cells among CD11c^+^ cells, the *TNF‐a*, *IL‐6*, and *IL‐12b* levels in the Aza‐DNBS NPs + US group were slightly greater than those in the Aza‐NH_2_ NPs + US group. These experimental findings suggest that Aza‐DNBS NPs have the capacity to release SO_2_, and amplify SDT under US irradiation, thereby enhancing ICD and subsequent antitumor immune responses.

### 2.4 Therapeutic Effect of Aza‐DNBS NPs‐Mediated SDT in Combination with PD‐L1 Blockade in a Mouse Model of Orthotopic Luciferase‐Transfected H22 cell (H22‐Luc) HCC

**Figure 3:**
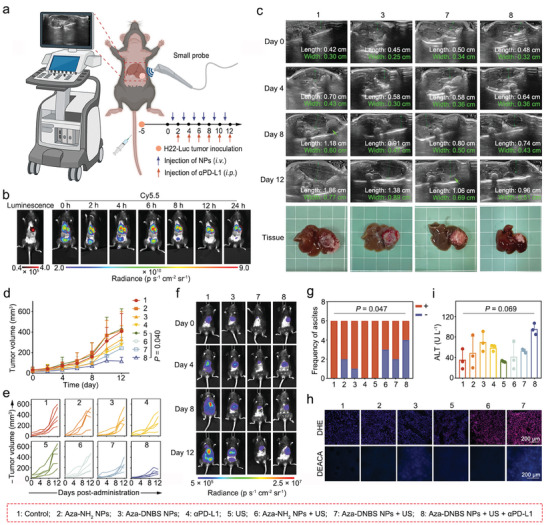
In vivo synergistic therapy involving PD‐L1 blockade and Aza‐DNBS NPs‐mediated SDT. a) Schematic diagram representing the in vivo treatment protocol for H22‐Luc orthotopic tumors. Mice with orthotopic H22‐Luc tumors were treated with Aza‐NH_2_ NPs or Aza‐DNBS NPs every 2 days after intravenous injection. Additionally, αPD‐L1 was administered to each mouse every 2 days at a dose of 100 µg by intraperitoneal injection. b) Representative IVIS images displaying the accumulation of Aza‐DNBS NPs within tumors at various time points postinjection. c) High‐frequency US images of the H22‐Luc orthotopic tumor‐bearing C57BL/6 mice receiving diverse treatments on days 0, 4, 8, and 12, along with the corresponding ex vivo tissue images. The green arrows mark the ascites. d) Mean tumor growth curves of each indicated treatment group (*n* = 5), with statistical significance, were calculated through the Kruskal–Wallis test. e) Individual tumor growth curves of each treatment group. f) Representative IVIS images of the H22‐Luc orthotopic tumor‐bearing C57BL/6 mice receiving various treatments on days 0, 4, 8, and 12. g) Comparative analysis of the occurrence of ascites in each group (*n* = 6) through the Chi‐Square test. h) Representative immunofluorescence images of ROS (in red) and SO_2_ (in blue) staining using DHE and DEACA as probes, respectively. i) Serum ALT analysis in different groups (*n* = 3), with statistical significance calculated through the Kruskal‐Wallis test.

Encouraged by the impressive outcomes of effective tumor elimination and BMDCs activation, we postulated that the combination of PD‐L1 blockade and Aza‐DNBS NPs‐mediated SDT could enhance the efficiency of the cancer‐immune cycle. This includes processes such as DCs maturation, T cell initiation, and immunogenic tumor cell death. Accordingly, we investigated the antitumor efficacy of Aza‐NH_2_ NPs/Aza‐DNBS NPs‐mediated SDT and the combined approach of Aza‐DNBS NPs‐mediated SDT and αPD‐L1 in the treatment of orthotopic HCC in vivo (**Figure** **3**a). In addition, we injected Cy5.5‐labeled Aza‐DNBS NPs to monitor the accumulation of Aza‐DNBS NPs within the tumor and determine the optimal time points for US treatment. As indicated in Figure 3b and Figure S11 (Supporting Information), Aza‐DNBS NPs began to accumulate in tumors at 2 h postadministration and peaked at 4 h. There was still some accumulation at 24 h after injection. Therefore, US irradiation was performed at 4 h after NPs injection, guided by high‐frequency US imaging.

Tumor progression was tracked using high‐frequency US imaging (Figure 3c; Figure S12, Supporting Information). Initially, we included 6 mice per group, but due to some deaths that occurred during the treatment, only tumor growth data from 5 mice per group were analyzed. The results showed the combination of Aza‐DNBS NPs‐mediated SDT and αPD‐L1 was significantly more effective than either Aza‐DNBS NPs + US treatment or αPD‐L1 treatment alone treatment alone (average tumor volumes are shown in Figure 3d, and individual growth curves are shown in Figure 3e). Additionally, we employed an in vivo imaging system (IVIS) to monitor the development of orthotopic tumors and similar results were obtained (Figure 3f; Figure S13, Supporting Information). Moreover, based on the ultrasonography and IVIS imaging results, the occurrence of ascites and peritoneal seeding metastasis was observed, especially in the control group. Pooled data indicated that the combination treatment delayed the onset of ascites and reduced its incidence compared to the control and US groups (Figure 3g; Figure S14a, Supporting Information). As shown in Figure S14b (Supporting Information), some mice, particularly those in the control and US groups, died during the treatment. In contrast, no deaths occurred in the Aza‐DNBS NPs + US + αPD‐L1 group. This may be attributed to the delayed onset of ascites, which is a critical factor affecting the prognosis of HCC patients.^[^ ^16^ ^]^ Consistent with the above results, hematoxylin and eosin (H&E), protein phosphatase 1 (Ki67), and terminal deoxynucleotidyl transferase‐mediated dUTP nick end labeling (TUNEL) staining images also revealed that Aza‐DNBS NPs + US + αPD‐L1 resulted in the lowest proliferation of tumor cells and the highest level of tumor cell death in tumor tissues (Figure S15, Supporting Information).

The antitumor mechanism of NPs under US irradiation was further investigated using dihydroethidium (DHE) and DEACA staining (Figure 3h). Compared to those of the other treatment groups, the DHE staining images of the Aza‐NH_2_ NPs + US and Aza‐DNBS NPs + US groups exhibited a strong red fluorescence signal, indicating efficient ROS generation. Additionally, the blue fluorescence intensities in the Aza‐DNBS NPs and Aza‐DNBS NPs + US groups markedly increased, verifying the effective release of SO_2_. Considering the high accumulation of NPs in normal liver tissues, we measured serum alanine aminotransferase (ALT) levels to assess the effect of NPs‐mediated SDT on normal liver tissues. As depicted in Figure 3i, compared to the other groups, the Aza‐DNBS NPs + US + αPD‐L1 group had a greater ALT level, ≈95 U L^−1^. The increase in ALT in the Aza‐DNBS NPs + US irradiation group was negligible. According to the guidelines of the European Society for Medical Oncology, mild liver injury caused by immunotherapy is acceptable if the ALT level is slightly elevated, but is still less than three times the upper limit of normal (120 U L^−1^).^[^ ^17^ ^]^ Moreover, we examined the major organs by H&E staining. The results revealed no discernible abnormalities or differences in the major organs or body weights of the treatment groups (Figures S16 and S17, Supporting Information). These findings further underscore the in vivo biosafety of Aza‐DNBS NPs‐mediated SDT and in combination therapy with αPD‐L1.

### 2.5 Antitumor Immune Responses Elicited by Aza‐DNBS NPs‐Mediated SDT Combined with PD‐L1 Blockade

**Figure 4:**
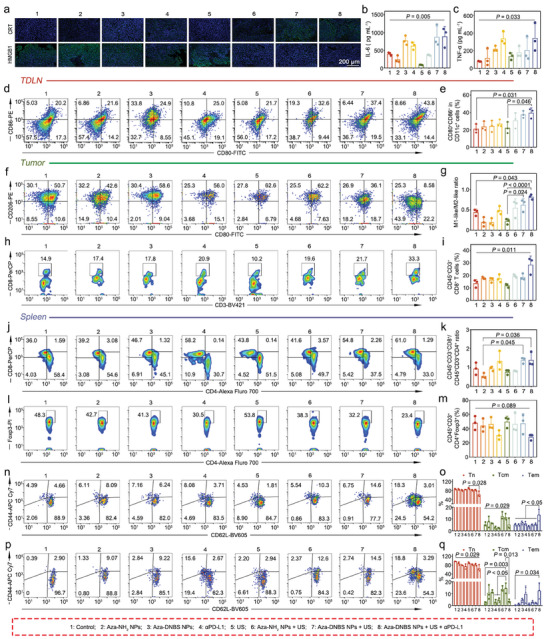
TME programming by Aza‐DNBS NPs‐mediated SDT and its synergistic therapy with PD‐L1 blockade. a) Immunofluorescence images showing CRT and HMGB1 in tumor tissues after various treatments. b,c) ELISA analysis of b) serum IL‐6 and c) TNF‐α (*n* = 3). d) Representative flow cytometry analysis and e) the corresponding statistical results indicating DC maturation (CD80^+^CD86^+^ proportion in CD11c^+^ cells) in the TDLN (*n* = 3). f) Flow cytometry analysis of TAMs in the TME. g) Statistical analysis of the M1‐like/M2‐like (F4/80^+^ CD80^high^CD206^low^/F4/80^+^CD80^low^CD206^high^) macrophage ratio (*n* = 3). h) Representative flow cytometry analysis of infiltrated CD8^+^ T cells (CD45^+^CD3^+^CD8^+^ cells) in the TME i) and the corresponding statistical results (*n* = 3). j) Representative flow cytometry analysis of CD8^+^ T cell (CD45^+^CD3^+^CD8^+^) and CD4^+^ T cell (CD45^+^CD3^+^CD4^+^) proportions in the spleen and k) the relative quantification of the proportion of CD8^+^/CD4^+^ T cells (*n* = 3). l,m) Flow cytometry analysis and m) the corresponding statistics representing the proportion of Tregs (CD45^+^CD3^+^CD4^+^Foxp3^+^ cells) in the spleen *n* = 3). n) Flow cytometry analysis and o) the corresponding statistical results showing the proportions of Tn (CD45^+^CD3^+^CD4^+^CD44^low^CD62L^high^ cells), Tcm (CD45^+^CD3^+^CD4^+^CD44^high^CD62L^high^ cells) and Tem (CD45^+^CD3^+^CD4^+^CD44^high^CD62L^low^ cells) in splenic CD4^+^ T cells (*n* = 3). p) Flow cytometry analysis and q) the corresponding statistical results illustrating the proportions of Tn (CD45^+^CD3^+^CD8^+^CD44^low^CD62L^high^ cells), Tcm (CD45^+^CD3^+^CD8^+^CD44^high^CD62L^high^ cells) and Tem (CD45^+^CD3^+^CD8^+^CD44^high^CD62L^low^ cells) in CD8^+^ T cells in the spleen (*n* = 3). All the statistical analyses were conducted using the Kruskal‐Wallis test to determine the significance.

Based on the hypothesis that Aza‐NH_2_ NPs/Aza‐DNBS NPs‐mediated SDT could induce ICD in tumor cells in vivo, we conducted immunohistochemistry (IHC) analysis to assess CRT and HMGB1 expression in orthotopic tumors. As depicted in **Figure** **4**a, CRT was mildly elevated in the Aza‐NH_2_ NPs + US group and substantially elevated after treatment with Aza‐DNBS NPs + US and the combination of Aza‐DNBS NPs‐mediated SDT and αPD‐L1. In contrast, HMGB1 expression significantly decreased in the Aza‐NH_2_ NPs + US, Aza‐DNBS NPs + US, and Aza‐DNBS NPs + US + αPD‐L1 groups. These results indicate that Aza‐NH_2_ NPs/Aza‐DNBS NPs‐mediated SDT can successfully induce ICD in orthotopic HCC. Furthermore, we examined the plasma levels of cytokine, including IL‐6 and TNF‐α, using enzyme‐linked immunosorbent assays (ELISA). The results revealed that the synergistic treatment with SDT mediated by Aza‐DNBS NPs and PD‐L1 blockade significantly upregulated IL‐6 and TNF‐α levels compared to those in the other groups, indicating immune cell activation (Figure 4b,c).

Therefore, we analyzed the innate and adaptive immune cell populations in the tumor‐draining lymph nodes (TDLN), TME, and spleen of H22‐Luc orthotopic HCC mice 2 days after treatment. Flow cytometry analysis revealed that the proportions of mature DCs in the TDLN (percentages of CD80^+^CD86^+^ in CD11c^+^ cells, Figure S18, Supporting Information) in the Aza‐DNBS NPs + US and Aza‐DNBS NPs + US + αPD‐L1‐treated mice were 1.85‐ and 2.16‐fold greater than those in the control group, respectively. This finding aligned with the enhancement of tumor ICD induced by Aza‐DNBS NPs‐mediated SDT and PD‐L1 blockade combination therapy (Figure 4d,e). Moreover, in the TME, we observed the proportions of tumor‐associated macrophages (TAMs), including M1‐like macrophages (CD80^high^CD206^low^ in F4/80^+^ cells) and M2‐like macrophages (CD80^low^CD206^high^ in F4/80^+^ cells, Figure S19, Supporting Information). As shown in Figure 4f,g, M2‐like macrophages repolarized to a more M1‐like state in the mice treated with Aza‐DNBS NPs + US alone or with ICB, and the ratio of the M1‐like percentage to the M2‐like percentage increased. Tumor T cell infiltration analysis revealed that the synergistic effect of Aza‐DNBS NPs‐mediated SDT and αPD‐L1 also obviously increased the infiltration of CD8^+^ T cells (CD45^+^CD3^+^CD8^+^ cells) in the TME, which was approximately twofold greater than that in the control group (Figure S19, Supporting Information; Figure 4h,i). In the spleen, similar results were observed. Compared with those in the control group, the proportion of CD8^+^ T cells in the Aza‐DNBS NPs + US treatment group or αPD‐L1 treatment group was increased. However, the combination therapy was significantly more effective than either treatment alone (Figure 4j). The ratio of CD8^+^/CD4^+^ T cells increased after treatment with Aza‐DNBS NPs + US alone or in combination with ICB (Figure 4k). The proportion of Tregs (CD45^+^CD3^+^CD4^+^Foxp3^+^ cells, Figure S20, Supporting Information), known for their immunosuppressive activity and importance in tumor immune evasion, decreased after synergistic therapy (Figure 4l,m). We further investigated whether T cell memory had formed in the spleen. The percentages of CD4^+^CD44^high^CD62L^high^ central memory T cells (Tcm) were increased in mice treated with Aza‐NH_2_ NPs + US and Aza‐DNBS NPs + US. In the Aza‐DNBS NPs + US + αPD‐L1 group, CD4^+^CD44^high^CD62L^low^ effector memory T cells (Tem) were elevated, while the native T cells (CD4^+^CD44^low^CD62L^high^, Tn) were downregulated. Interestingly, the combination with ICB increased the percentage of CD4^+^ Tem but decreased the percentage of CD4^+^ Tcm compared to those in the Aza‐DNBS NPs + US group, suggesting a transformation from Tcm to Tem (Figure 4n,o). A similar profile was found for CD8^+^ T cells, where the Aza‐DNBS NPs + US‐treated mice had a greater percentage of CD8^+^ Tcm, and combination treatment with anti‐PD‐L1 resulted in a higher percentage of CD8^+^ Tem cells. An increasing trend in CD8^+^ Tem was also observed in the mice treated with αPD‐L1 alone (Figure 4p,q). These findings provide evidence that both Aza‐DNBS NPs + US treatment and combination therapy with ICB can effectively promote the production of immune memory cell populations and that synergistic treatment facilitates the transformation of Tcm to Tem, contributing to the observed differences in antitumor efficiency between these treatments.

### 2.6 Lung Metastasis Inhibition Elicited by Combination Treatment

**Figure 5:**
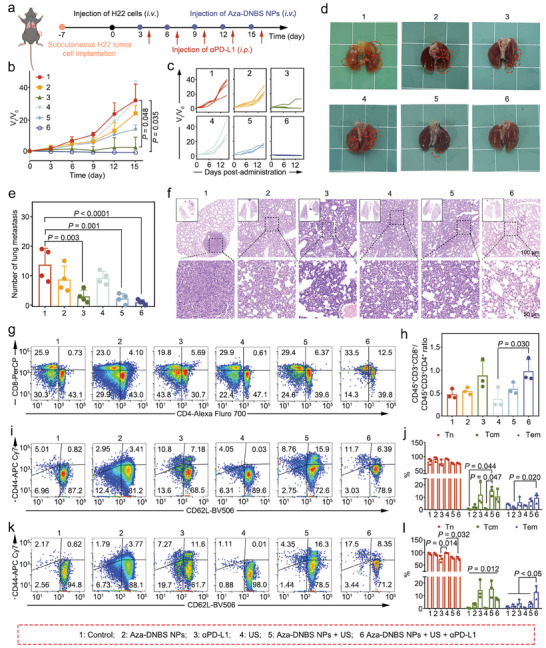
Assessment of the therapeutic efficacy of Aza‐DNBS NPs‐mediated SDT with αPD‐L1 therapy in an H22 pulmonary metastasis model. a) Schematic diagram of the establishment of the lung metastatic mouse model and administration schedule. b) Changes in H22 subcutaneous tumor volumes throughout the treatment period (*n* = 4). c) Individual tumor growth curves of each indicated treatment group. d) Representative images of lungs from each group. e) The number of pulmonary nodules was counted (*n* = 4). f) Representative H&E staining images of lungs at the end of the treatment. g) Representative flow cytometry analysis of the proportions of CD8^+^ T cells and CD4^+^ T cells in the blood, and h) the relative quantification of the CD8^+^/CD4^+^ T cell ratios (*n* = 3). i) Flow cytometry analysis images and j) the corresponding statistics denoting the proportions of Tn (CD45^+^CD3^+^CD4^+^CD44^low^CD62L^high^ cells), Tcm (CD45^+^CD3^+^CD4^+^CD44^high^CD62L^high^ cells) and Tem (CD45^+^CD3^+^CD4^+^CD44^high^CD62L^low^ cells) in blood CD4^+^ T cells (*n* = 3). k) Flow cytometry analysis and l) the corresponding statistical data depicting the proportions of Tn (CD45^+^CD3^+^CD8^+^CD44^low^CD62L^high^ cells), Tcm (CD45^+^CD3^+^CD8^+^CD44^high^CD62L^high^ cells) and Tem (CD45^+^CD3^+^CD8^+^CD44^high^CD62L^low^ cells) in CD8^+^ T cells in the blood (*n* = 3). All the statistical analyses were conducted using the Kruskal‐Wallis test to determine the significance.

Distant tumor metastasis remains the major roadblock for successful clinical treatment.^[^ ^18^ ^]^ Encouraged by the observed systemic immune activation, we next investigated the potential of our therapeutics in preventing lung metastasis of aggressive HCC (**Figure** **5**a). The growth curve of subcutaneous tumors showed that both αPD‐L1 and combination therapy significantly inhibited tumor growth and essentially eliminated tumors. Aza‐DNBS NPs‐ mediated SDT also displayed a certain tumor‐suppressive effect (Figure 5b,c; Figure S21, Supporting Information). Analysis of lung metastasis revealed that Aza‐DNBS NPs alone were not effective in inhibiting the lung metastasis of HCC. Both Aza‐DNBS NPs + US and αPD‐L1 could evidently suppress lung metastasis, and the synergistic treatment with Aza‐DNBS NPs + US and αPD‐L1 manifested better efficiency (Figure 5d,e). H&E staining of lung tissues also revealed that compared with the other treatment groups, the Aza‐DNBS + US + αPD‐L1 group exhibited markedly fewer metastatic tumor lesions (Figure 5f).

To further understand the mechanisms underlying this therapeutic effect, we studied the T cell populations in the blood samples collected from different groups. We found a greater CD8^+^/CD4^+^ ratio in the Aza‐DNBS NPs + US + αPD‐L1 group (Figure 5g,h). Notably, the percentages of CD4^+^ Tcm and CD8^+^ Tcm were markedly greater in the Aza‐DNBS NPs + US/αPD‐L1 therapy alone group than in the combination therapy group. Moreover, αPD‐L1 treatment and combination therapy could facilitate the transformation of Tcm to Tem, with an increase in the proportion of CD4^+^ Tem and CD8^+^ Tem, and the combination treatment group was more effective than the αPD‐L1 group (Figure 5i–l). These results suggest that the robust systemic immune response activation triggered by Aza‐DNBS NPs + US/αPD‐L1 alone could inhibit the occurrence of aggressive HCC lung metastasis, to some extent, and that combination therapy with Aza‐DNBS NPs + US & αPD‐L1 had a more effective therapeutic effect. The minimal change in body weight throughout the treatment also confirmed the biosafety of the various treatments (Figure S22, Supporting Information).

### 2.7 Immunosuppressive TME Reprogramming Induced by Aza‐DNBS NPs‐Mediated Enhanced SDT

**Figure 6:**
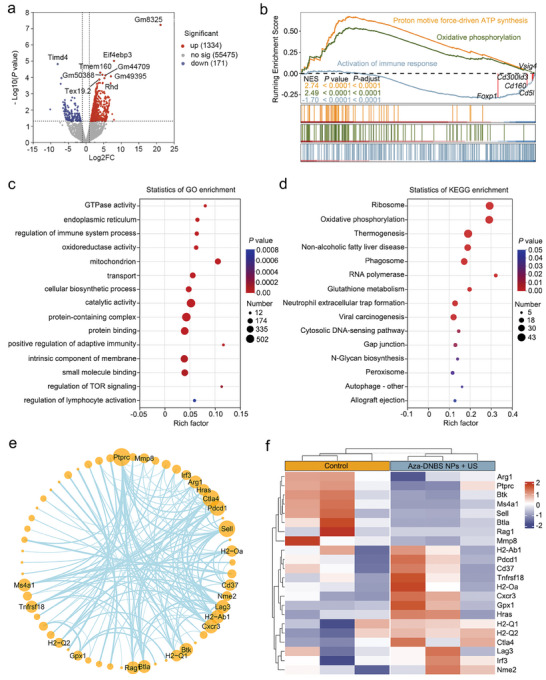
Transcriptome sequencing analysis exploring the mechanism underlying the programming of the TME by Aza‐DNBS NPs‐mediated SDT. a) Volcano plot of DEGs. Each point represents a gene: the *x*‐axis corresponds to the log twofold change in the ratio, while the y‐axis signifies the probability that a gene demonstrates statistical significance in its differential expression. b) Representative pathways enriched in the identified genes as determined by GSEA. c) GO and d) KEGG enrichment analysis of the DEGs. e) Protein‐protein interaction network related to the regulation of immune system processes and f) the heatmap of the expression of DEGs involved.

Current studies have confirmed the capacity of SDT to regulate the TME only at the cellular level, as described above, and the underlying molecular mechanisms remain elusive.^[^ ^19^ ^]^ Therefore, we extracted immune cells from H22‐Luc orthotopic tumors in the control and Aza‐DNBS NPs + US groups (*n* = 3 in each group) and conducted transcriptome sequencing analysis to determine the underlying mechanisms involved. Principal component analysis (PCA) was used to determine the distinct distribution between the two groups according to the whole genome, and the results showed that the distribution maps of the first two principal components determined by PCA corresponded to 67.88% and 19.5% of the cumulative variance contribution rate, respectively (Figure S23, Supporting Information). A total of 1334 differentially expressed genes (DEGs) were significantly upregulated and 171 DEGs were distinctly downregulated in the comparison of the two groups (**Figure** **6**a). Among the most significant DEGs, *Timd4*, which is known to impair CD8^+^ T cell proliferation when highly expressed in macrophages thereby affecting ICB efficacy,^[^ ^20^ ^]^ was particularly noteworthy. Similarly, *Eif4ebp3*, a marker for long‐term inhibition of mechanistic/mammalian target rapamycin complex 1 (mTORC1), a promising cancer treatment strategy,^[^ ^21^ ^]^ was also markedly affected. The downregulation of *Timd4* and the upregulation of *Eif4ebp3* after treatment with Aza‐DNBS NPs + US suggest that Aza‐DNBS NPs + US are involved in this mechanism of tumor immunomodulation.

By conducting gene set enrichment analysis (GSEA), we determined that the Aza‐DNBS NPs + US group was negatively correlated with the “activation of immune response” dataset, in which negative regulators of antitumor immunity, including *Foxp1*,^[^ ^22^ ^]^ *Cd5l*,^[^ ^23^ ^]^ *Cd160*,^[^ ^24^ ^]^ *Cd300ld3*,^[^ ^25^ ^]^ and *Vsig4* ^[^ ^26^ ^]^ were the core genes. In contrast, the Aza‐DNBS NPs + US group was positively regulated by mitochondrial metabolic processes, such as proton motive force‐driven ATP synthesis and oxidative phosphorylation (Figure 6b), which are important for the regulation of mitochondria in the immune cells.^[^ ^27^ ^]^ Furthermore, Gene Ontology (GO) and Kyoto Encyclopedia of Genes and Genomes (KEGG) enrichment analyses revealed that DEGs were associated with immune regulation and mitochondrial metabolism (Figure 6c,d). To further elucidate the alterations in protein interactions involved in immune system response regulation, we identified 22 interacting hub genes based on modality using the STRING database (Figure 6e). Notably, the genes *Ctla4* and *Cd37* were upregulated after treatment with Aza‐DNBS NPs + US, while *Btla* was downregulated (Figure 6f). These changes indicate T cell and B cell maturation, implying that the combination treatment of Aza‐DNBS NPs + US with anti‐CTLA4 therapy may hold significant promise.^[^ ^28^ ^]^ These findings suggest that Aza‐DNBS NPs + US treatment induces the activation of the adaptive immune response within the TME. Regarding innate immunity, previous studies have shown that tumor cell ICD can release double‐stranded DNA (dsDNA) that can be taken up by macrophages or DCs,^[^ ^29^ ^]^ which is consistent with our results.

Figure 6d shows that the DEGs were enriched in the cytosolic DNA‐sensing pathway and phagosomes. Among the phagosome‐related genes, major histocompatibility complex (MHC) class I and II protein complex‐related genes (*H2‐Q1*, *H2‐Q2*; *H2‐Oa*, *H2‐Ab1*, *H2‐DMa*) were upregulated, suggesting that the antigen presentation occurs in antigen‐presenting cells (APCs, Figure S24, Supporting Information).^[^ ^30^ ^]^ Additionally, we screened 275 genes expressed exclusively in the Aza‐DNBS NPs + US group and performed GO enrichment analysis. The results revealed that genes related to regulatory activation of B cells, complement activation, and immunoglobulin were primarily enriched (Figure S25, Supporting Information). Based on the above results, we suggest that the ICD of tumor cells induced by Aza‐DNBS NPs + US can release dsDNA and other DAMPs. They are phagocytosed and recognized by APCs, simultaneously activating the complement system, promoting the phagocytosis and antigen presentation ability of APCs, and thereby activating T cell‐ and B cell‐mediated adaptive immunity.^[^ ^31^ ^]^ This profound reprogramming enhances the efficacy of αPD‐L1, achieving superior therapeutic efficacy for HCC and HCC lung metastasis aforementioned.

## 3 Conclusion

Efforts to enhance the efficacy of SDT and amplify the immune responses induced by SDT have become a research focus in cancer therapy. In this vein, we used SO_2_ to enhance ROS production and constructed SO_2_ prodrug‐loaded nanoparticles, Aza‐DNBS NPs, to improve the SDT effect. In vitro studies and orthotopic H22‐Luc models demonstrated that the ICD of tumor cells induced by Aza‐DNBS NPs + US, in conjunction with αPD‐L1, effectively elicited systemic immune responses and significantly suppressed orthotopic tumor growth and lung metastasis.

To elucidate the mechanisms underlying the enhanced SDT‐elicited immune responses mediated by the Aza‐DNBS NPs, we harvested immune cells from orthotopic H22‐Luc tumors and performed transcriptome sequencing. Our findings suggested that SDT‐induced ICD in tumor cells releases dsDNA and other DAMPs, which are likely to be absorbed by APCs and activate the cytosolic dsDNA‐sensing signaling pathway. This activation subsequently upregulated MHC I and MHC II molecule‐related genes to enhance antigen presentation by APCs. Concurrently, immunosuppression‐related genes, including *Timd4*, *Foxp1*, *Cd5l*, *Cd160*, *Cd300ld3*, and *Vsig4*, were downregulated and stimulated the pathways related to the activation of T cells and B cells. This multifaceted process effectively reprogrammed the immunosuppressive TME. We believe that the favorable efficacy of Aza‐DNBS NPs + US + αPD‐L1 in the treatment of orthotopic HCC tumors and distant lung metastasis can be attributed to the reprogramming of the immune microenvironment. Therefore, synergistic therapy involving Aza‐DNBS NP‐mediated enhanced SDT combined with ICB shows significant promise for potential clinical applications in cancer treatment.

## 4 Experimental Section

### Fabrication of Aza‐NH_2_ NPs and Aza‐DNBS NPs

The synthesis of Aza is detailed in the supplementary information. To create Aza‐NH_2_ NPs, Aza (10 mg) and DSPE‐PEG‐NH_2_ (50 mg) were added to chloroform (50 mL), and the mixture was stirred for 24 h at room temperature. To produce DSPE‐PEG‐DNBS, DNBS (5.33 mg, 0.02 mmol), DSPE‐PEG‐NH_2_ (40 mg, 0.02 mmol), and NaHCO_3_ (1.68 mg, 0.02 mmol) were added to tetrahydrofuran (20 mL) and stirred for 8 h at room temperature. After rotational evaporation and washing, DSPE‐PEG‐DNBS (50 mg) and Aza (10 mg) were added to chloroform (50 mL) and stirred for 24 h at room temperature. The solvent was then removed, resulting in the final Aza‐DNBS NPs.

### Characterization of the NPs

The sizes and zeta potentials of the Aza‐NH_2_ NPs and Aza‐DNBS NPs were measured by DLS. TEM was utilized to characterize their morphologies and sizes. FTIR spectra of Aza, Aza‐NH_2_ NPs, and Aza‐DNBS NPs were recorded by a VERTEX70 V FTIR spectrometer. To verify the GSH‐responsive SO_2_ release capacity of Aza‐DNBS NPs, the NPs were resuspended in PBS (pH 5.8) containing 10 µm GSH and 5 µm DEACA. Then, the fluorescence intensities at different time points were measured by a Varioskan LUX multifunctional microplate reader (λ_ex_ = 390 nm, λ_em_ = 483.5 nm). DPBF (10 mmol L^−1^ in ethanol solution) was used as the ^1^O_2_ indicator to detect the ^1^O_2_ generation. The diverse solutions were exposed to US irradiation (1.0 W cm^−2^) for 5 min, respectively. For ESR spectroscopy measurements, the ^1^O_2_ generation was detected using TEMP as a ^1^O_2_ trapping agent.

### In Vitro SDT Mediated by NPs and ICD Induction of Tumor Cells

First, the cellular uptake of Aza‐DNBS NPs in Huh7 cells was assessed by bio‐TEM. To assess the effectiveness of SDT mediated by NPs, 1 × 10^5^ Huh7 cells were seeded in six‐well dishes and subjected to various treatments: control, Aza‐NH_2_ NPs (100 µg mL^−1^), and Aza‐DNBS NPs (100 µg mL^−1^) for 6 h. The cells in the US, Aza‐NH_2_ NPs + US, and Aza‐DNBS NPs + US groups were then exposed to US irradiation (1.0 MHz, 1.0 W cm^−2^, 50% duty cycle, 3 min). Subsequently, CLSM was employed to visualize the intracellular ROS production and SO_2_ release using the probes DCFH‐DA and DEACA, respectively. In addition, intracellular GSH levels in Huh7 cells after various treatments were examined using GSH and GSSG assay kits. To assess the antitumor effect of NPs‐mediated SDT, Huh7 cells were collected at 24 h after different treatments. The calcein‐AM/PI and CCK‐8 assays were then performed following the provided protocol. Additionally, H22 cells in different treatment groups were stained with annexin V‐FITC and PI for flow cytometry analysis. Immunofluorescence was used to analyze the expression of ICD markers, including HMGB1 and CRT. Specifically, the treated Huh7 cells were then stained with an anti‐HMGB1 antibody, Alexa Fluor 647 conjugated rabbit anti‐goat IgG, and an anti‐CRT antibody, and Alexa Fluor 488 conjugated rabbit antigoat IgG, respectively. CLSM was used to assess changes in the immunofluorescence in various groups. Further fluorescence intensity analysis was performed using ImageJ software. Additionally, the expression of CRT on dead cell membranes (PI^+^ cells) was further detected by PI and CRT staining using flow cytometry, and the HMGB1 level released from Huh7 cells into the culture medium after various treatments was assessed using ELISA. Moreover, intracellular and extracellular ATP levels in Huh7 cells were assessed using an ATP assay kit.

### BMDCs Isolation and Maturation Assessment

Bone marrow was harvested by isolating and flushing the tibias and femurs of male C57BL/6 mice aged 6–8 weeks. Bone marrow cells, totaling 2 × 10^6^, were cultured in a complete medium infused with recombinant mouse granulocyte‐macrophage colony‐stimulating factor (20 ng mL^−1^) to generate BMDCs. Half of the medium was replaced on the third and fifth days. On day 6, the BMDCs were collected by simple washing and then cocultured with H22 cells after various pretreatments. The next day, the BMDCs were collected for maturation evaluation via flow cytometry and quantitative real‐time PCR (qPCR). For flow cytometry analysis, the following antibodies were used: FITC‐conjugated anti‐CD80, PE‐conjugated anti‐CD86, and APC‐conjugated anti‐CD11c. For qPCR, the primer sequences for the detected genes were as follows: *β‐actin* (5′‐GCACCCAGCACAATGAAGAT‐3′ and 5′‐ACATCTGCTGGAAGGTGGAC‐3′), *TNF‐a* (5′‐ CCCTCACACTCAGATCATCTTCT‐3′ and 5′‐ GCTACGACGTGGGCTACAG‐3′), *IL‐6* (5′‐ TAGTCCTTCCTACCCCAATTTCC‐3′ and 5′‐ TTGGTCCTTAGCCACTCCTTC‐3′), and *IL‐12b* (5′‐ TGGTTTGCCATCGTTTTGCTG‐3′ and 5′‐ ACAGGTGAGGTTCACTGTTTCT‐3′). *β‐actin* served as an internal control gene.

### H22‐Luc Orthotopic Model Establishment

To establish the H22‐Luc orthotopic model, ≈1 × 10^7^ H22‐Luc cells mixed with Matrigel (at a 2:1 v/v ratio) were implanted into the left extrahepatic lobe of C57BL/6 mice (female, 6–8 weeks old). High‐frequency ultrasonography was performed every 2 days to track tumor growth. Once the diameter of the orthotopic tumors reached ≈5 mm, the mice were randomly assigned to different treatment groups. The animal experiment was approved by the Ethics Committee of Shanghai University (ECSHU‐2022‐050).

### Biodistribution of Aza‐DNBS NPs in the H22‐Luc HCC Tumor Model

To investigate the distribution of Aza‐DNBS NPs in mice, Cy5.5‐labeled Aza‐DNBS NPs were injected intravenously. The IVIS imaging spectroscopy (λ_ex_ = 675 nm, λ_em_ = 720 nm) was utilized to capture the distribution images of Aza‐DNBS NPs in tumors at various time points postinjection (0, 2, 4, 6, 8, 12, and 24 h).

### In Vivo Therapeutic Effect of Aza‐DNBS NPs‐Mediated SDT in Combination with αPD‐L1

To evaluate the therapeutic effect of NPs‐mediated SDT and the combined antitumor effect of Aza‐DNBS NPs + US combined with anti‐PD‐L1 in an H22‐Luc orthotopic tumor model. The treatment protocol used is shown in Figure 4a. Mice with established orthotopic H22‐Luc tumors were administered with Aza‐NH_2_ NPs or Aza‐DNBS NPs at a dose of 500 µg kg^−1^ via intravenous (*i.v*.) injection, or αPD‐L1 by intraperitoneal (*i.p*.) injection (100 µg), which was repeated every 2 days for six cycles. Under the guidance of high‐frequency US imaging, US irradiation (1.5 MHz, 1.0 W cm^−2^, 50% duty cycle, 3 min) was applied at 4 h postinjection. To evaluate the therapeutic efficacy, tumor sizes in each treatment group (*n* = 5) were assessed every 2 days (days 0, 2, 4, 6, 8, 10, and 12) using high‐frequency US imaging. Tumor growth was also monitored by bioluminescence imaging in vivo. Specifically, bioluminescence images were recorded 8 min after intraperitoneal injection of 150 mg kg^−1^ D‐luciferin substrate on days 0, 2, 4, 6, 8, 10, and 12. Two days after the last treatment, all the mice were sacrificed. Main organs (heart, liver, lung, kidney, and spleen), tumor tissues, abdominal lymph nodes, and blood samples were collected for subsequent experiments.

### Pulmonary Metastasis Model Establishment and Treatment Strategy

A pulmonary metastasis model of liver cancer was established using mice bearing subcutaneous H22 tumors. Initially, the mice were injected subcutaneously with 5 × 10^5^ H22 cells. After a 7‐day period, the mice were intravenously injected with 1 × 10^6^ H22 cells, after which they were randomly allocated into different treatment groups (*n* = 4). The treatments were administered according to the schedule shown in Figure 5a. After 15 days of treatment (five times), the mice were sacrificed, and their lungs and blood samples were collected for subsequent analysis.

### Immunophenotyping Analysis

The collected tumor tissues were digested in 200 U mL^−1^ collagenase D and 15 U mL^−1^ DNase I in D‐Hanks balanced salt solution at 37 °C for 30 min to obtain the cell suspensions. Spleen and lymph node tissues were finely cut and ground to yield cell suspensions. The dissociated cells were then sieved through a 70 µm nylon cell strainer and collected for subsequent analyses. For blood samples, cells were obtained via centrifugation, and red blood cells were eliminated using erythrocyte lysates. The isolated cells were fixed and permeabilized for fluorescent probe entry for flow cytometry analysis. The fluorescent probes used and the detailed protocols used are shown in Figures S16–S18 (Supporting Information). During the analyses, fluorescence minus one (FMO) controls were used to establish the cutoff point. All analyses were conducted using FlowJo software.

### Cytokine and Biochemical Index Assays

At the end of the observation period, blood samples were collected from the mice that received various treatments. Plasma was then obtained by centrifugation. Subsequently, the levels of secreted IL‐6 and TNF‐α were determined using ELISA kits following the manufacturer's guidelines. In addition, serum ALT levels were assessed using an automated biochemistry analyzer.

### Histological Analysis and Immunohistochemical Staining

For histological analysis, tumor tissues and major organs from representative mice were excised and fixed in 4% paraformaldehyde. Both tumor and organ sections were stained with H&E. Cellular apoptosis after various treatments were evaluated by TUNEL and Ki67 staining of tumors. To assess the ROS generation and SO_2_ release in tumor tissues, DHE and DEACA immunofluorescence staining were conducted. Additionally, the expression of HMGB1 and CRT in tumors was assessed using anti‐HMGB1 and anti‐CRT antibodies, respectively.

### Transcriptome Sequencing Analysis of Immune Cells Extracted From the TME

Transcriptome sequencing analysis was performed by Shanghai Majorbio Bio‐pharm Technology Co., Ltd. Total RNA was isolated from immune cells from orthotopic H22‐Luc tumors in the control and Aza‐DNBS NPs + US groups by density‐gradient centrifugation (*n* = 3) for subsequent analysis. The analysis of DEGs (|log 2‐fold change| ≥ 1 and *p* < 0.05) was carried out based on the specified comparison groups. For GSEA analysis, the GseaVis R package was used. For GO and KEGG pathway analyses, the clusterProfiler R package was used.

### Statistical Analysis

Statistical analyses were carried out using IBM SPSS software version 23.0 and GraphPad Prism software version 8.0. One‐way ANOVA, the Kruskal‐Wallis test, and the Chi‐Square test were used to analyze the significance of differences between multiple groups. *P *< 0.05 was considered to indicate statistical significance.

## Conflict of Interest

The authors declare no conflict of interest.

## Supporting information



Supporting Information

## Data Availability

The data that support the findings of this study are available from the corresponding author upon reasonable request.
