# Supplementary material for: Activatable Sulfur Dioxide Nanosonosensitizer Enables Precisely Controllable Sono‐Gaseous Checkpoint Trimodal Therapy for Orthotopic Hepatocellular Carcinoma
Source: Adv Sci (Weinh). 2024 Dec 16;12(5):2409442. doi: 10.1002/advs.202409442 (PMC11791957; doi:10.1002/advs.202409442)
Supplement: Supplementary file 1 — Supporting Information [file ADVS-12-2409442-s001.docx]

Supporting Information

Activatable Sulfur Dioxide Nanosonosensitizer Enables Precisely Controllable Sono-Gaseous Checkpoint Trimodal Therapy for Orthotopic Hepatocellular Carcinoma

Jing Liang^‡^, Guangwen Cheng^‡^, Luping Qiu, Liyun Xue, Huning Xu, Xiaohui Qiao, Na Guo, Huijing Xiang*, Yu Chen*, and Hong Ding*

**Experimental Section**

Materials: Sodium hydroxide (NaOH)，ethanol (EtOH), benzaldehyde, 4-aminoacetophenone, hydrochloric acid (HCl), dichloromethane (CH_2_Cl_2_), potassium hydroxide (KOH), ethyl acetate (EtOAc), tetrahydrofuran, nitromethane, hexane, ammonium acetate, chalcone, triethylamine, boron trifluoride diethyl etherate, 2,4-dinitrobenzenesulfonyl chloride (DNBS), glutathione (GSH), and 7-diethylaminocoumarin-3-aldehyde (DEACA) were acquired from Chengdu Huaxia Reagent Co., Ltd (Chengdu, China). NaHCO_3_, DSPE-PEG-amine (DSPE-PEG-NH_2_), 1,3-diphenylisobenzofuran (DPBF), 2,2,6,6-tetramethylpiperidine (TEMP), and D-luciferin substrate were purchased from Shanghai Aladdin Biochemical Technology Co., Ltd (Shanghai, China). FBS, RPMI 1640 medium, Penicillin-Streptomycin, 0.25% trypsin-ethylene diamine tetraacetic acid (EDTA) solution, Matrigel, TriPure reagent, and M-MLV Reverse Transcriptase kit were purchased from Thermo Fisher Scientific (Massachusetts, USA). Annexin V-FITC apoptosis detection kit, calcein acetoxymethyl ester/propidium iodide (calcein-AM/PI), cell counting kit-8 (CCK-8), 2,7-dichlorodihydrofluorescein diacetate (DCFH-DA) assay kit, ATP assay kit, GSH and GSSG assay kit were obtained from Beyotime Biotechnology (Shanghai, China). (Invitrogen, USA). SYBR-Green premix was obtained from Roche Diagnostic (Mannheim, Germany). Anti-mouse PD-L1 was purchased from BioCell (New Hampshire, USA). Tumor necrosis factor-alpha (TNF-a), interleukin 6 (IL-6) enzyme-linked immunosorbent assays (ELISA), rabbit recombinant antibody against calreticulin (CRT), rabbit polyclonal monoclonal antibody against high mobility group box 1 (HMGB1), Alexa Fluor 488 conjugated rabbit anti-goat IgG, and Alexa Fluor 647 conjugated rabbit anti-goat IgG were purchased from Proteintech Group (Wuhan, China). FITC-conjugated anti-CD80, PE-conjugated anti-CD86, APC-conjugated anti-CD11c, PE-conjugated anti-CD206, APC-conjugated anti-F4/80, BV510-conjugated anti-CD45, BV421-conjugated anti-CD3, PI-conjugated anti-Foxp3, APC Cy7-conjugated anti-CD44, and BV605-conjugated anti-CD62L antibodies were purchased from Thermo Fisher Scientific (Massachusetts, USA). Alexa Fluro 700-conjugated anti-CD4, and PerCP-conjugated anti-CD8 antibodies were purchased from BioLegend (San Diego, USA).

Cell line and animal: The human normal hepatocellular cell line, L-02 cells and murine hepatocellular carcinoma cell lines, H22 and luciferase-transfected H22 cell (H22-Luc) were purchased from Shanghai Fuheng Biotechnology Co., Ltd (Shanghai, China). The human hepatocellular carcinoma cell line Huh7 was purchased from the Cell Bank of the Chinese Academy of Sciences. C57BL/6 were purchased from Gempharmatech Co., Ltd (Nanjing, China).

Synthesis of sonosensitizer azadipyrromethene (Aza): First, a stirred solution of NaOH (1.2 g, 0.03 mol) in EtOH (10 mL) and distilled water (10 mL) was prepared. Subsequently, 4-aminoacetophenone (1.35 g, 10 mmol) and benzaldehyde (1.06 g, 10 mmol) were added. The mixture was then heated under reflux for 6 h. The solution was acidified with dilute HCl and then partitioned between EtOAc and water. The organic layer was subsequently evaporated to dryness, and the resulting residue was purified using column chromatography with CH_2_Cl_2_ as the eluent. The resulting yellow solid, known as compound 1. Next, compound 1 (1 g, 4.46 mmol) was dissolved in EtOH (15 mL), followed by the addition of KOH (0.3 g, 5.35 mmol) and nitromethane (2.86 g, 46.87 mmol). The resulting mixture was heated under reflux for 12 h. After this, the solution was partitioned and the organic layer was evaporated to dryness. The resulting residue was purified by column chromatography using a hexane/EtOAc (at a 2:3 v/v ratio) mixture as the eluent. The obtained oily brown product was identified as compound 2. Subsequently, compound 2 (0.57 g, 2.45 mmol) and ammonium acetate (7.32 g, 95 mmol) were dissolved in EtOH (20 mL), and the mixture was heated under reflux for 6 h. After heating, the solution was partitioned, and the organic layer was evaporated to dryness. The resulting residue was purified by column chromatography using an EtOAc/CH_2_Cl_2_ (at a 1:6 v/v ratio) mixture as the eluent. The resulting blue-green crystals were identified as compound 3. Finally, compound 3 (0.31 g, 0.65 mmol), triethylamine (2 mL), and boron trifluoride diethyl etherate (2 mL) were dissolved in CH_2_Cl_2_ (30 mL). The mixture was then heated at 40 °C under a nitrogen atmosphere for 12 h. The solution was then partitioned and the residue obtained after rotational evaporation was purified by column chromatography using a hexane/EtOAc (at a 1:1 v/v ratio) mixture as the eluent. The obtained blue-green crystals obtained were identified as Aza. ^1^H NMR (400 MHz, Chloroform-d) δ 8.04 (dd, J = 16.6, 7.9 Hz, 10H), 7.49–7.36 (m, 8H), 7.05 (s, 2H), 6.75 (d, J = 8.7 Hz, 4H).

**Supplementary Figure Legends**


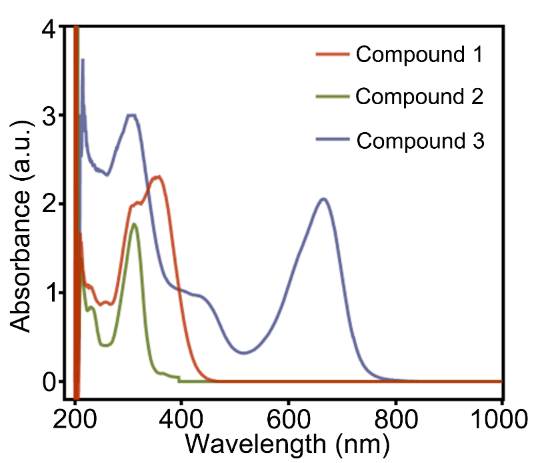


**Figure S1.** UV/Vis absorption spectra of the intermediates dissolved in EtOH.


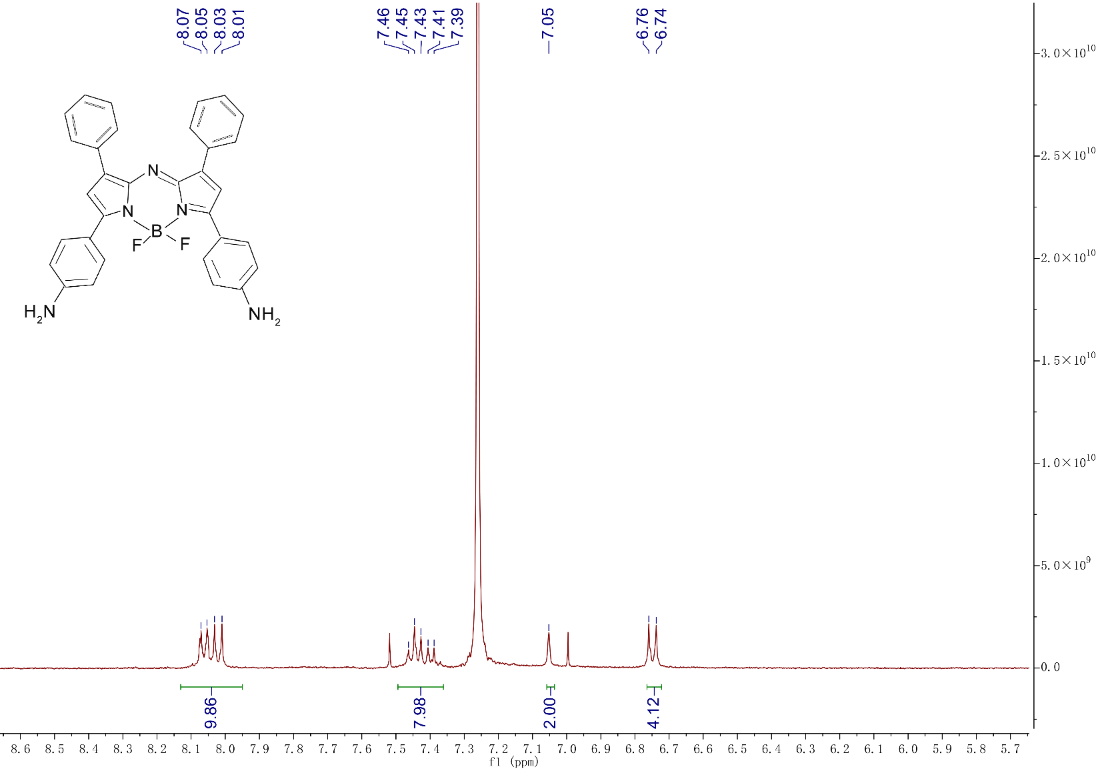


**Figure S2.** ^1^H NMR spectrum of Aza (400 MHz, Chloroform-d).


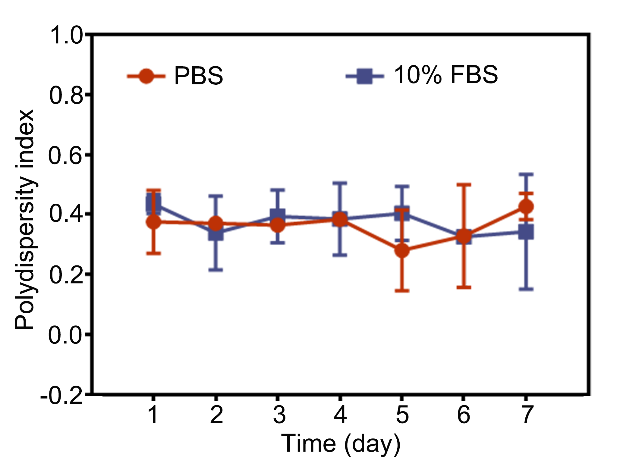


**Figure S3.** The variation of polydispersity index of the Aza-DNBS NPs, dissolved in PBS and PBS supplemented with 10% foetal bovine serum (FBS) over 7 days.


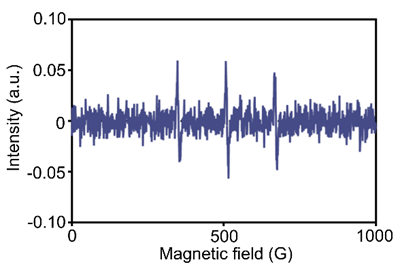


**Figure S4.** Electron spin resonance (ESR) spectroscopy spectra of PBS + US group using 2,2,6,6-tetramethylpiperidine (TEMP) as a ^1^O_2_ capturing agent.


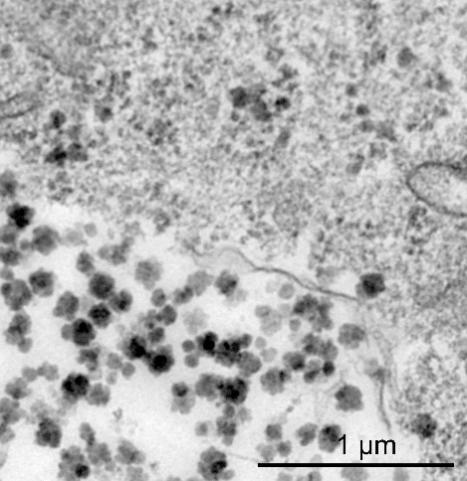


**Figure S5.** Bio-TEM image depicting the intracellular uptake of Aza-DNBS NPs.


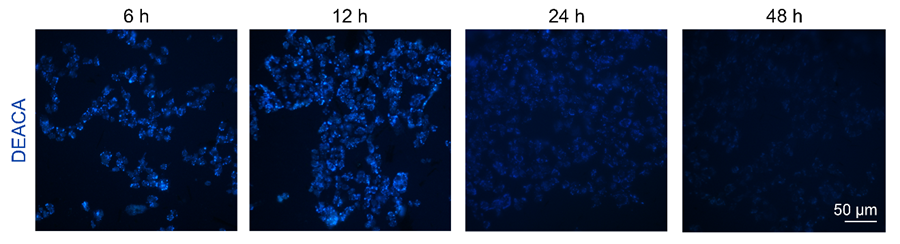


**Figure S6.** Representative immunofluorescence images of SO_2_ detection using DEACA as a probe after incubation of Aza-DNBS NPs with Huh7 cells over various time periods.


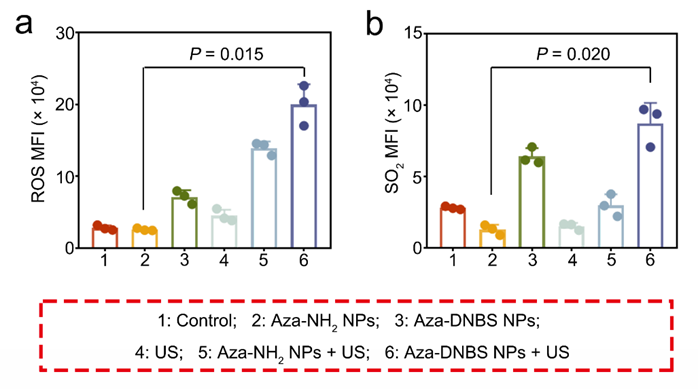


**Figure S7.** Quantitative results reflecting the mean fluorescence intensities of (a) ROS and (b) SO_2_ after various treatments. Statistical significance was calculated through the Kruskal-Wallis test.


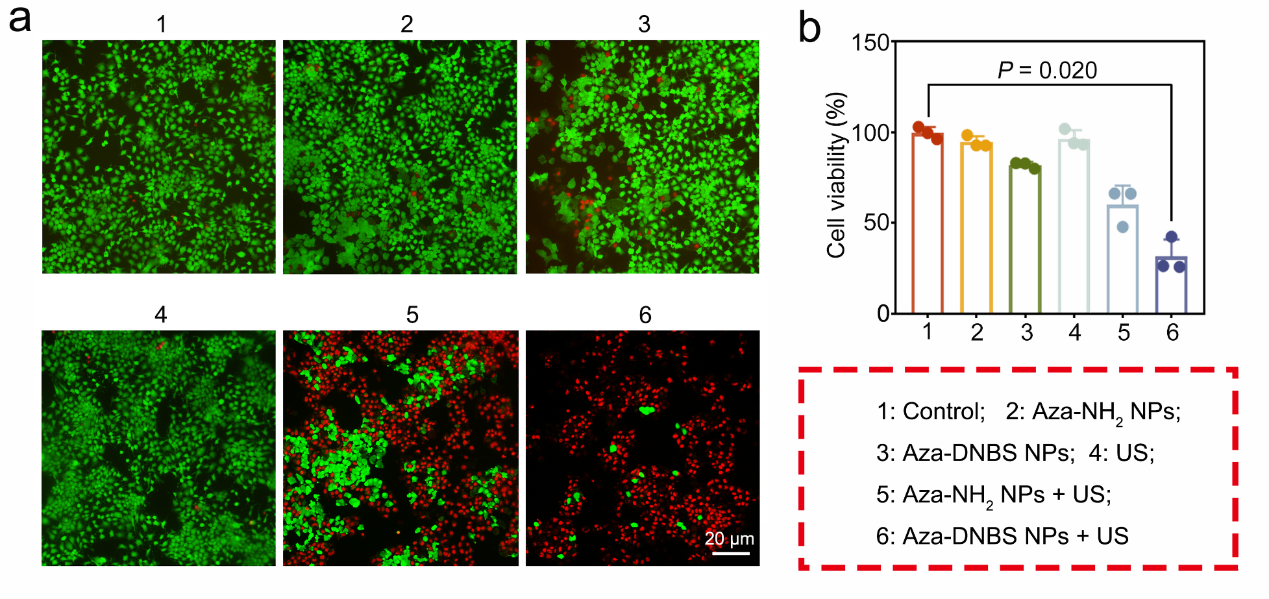


**Figure S8.** (a) Calcein-AM/PI analysis of the cytotoxic effects of NPs on Huh7 cells. (b) The viability rates of Huh7 cells after different treatments. Statistical significance was calculated through the Kruskal-Wallis test.


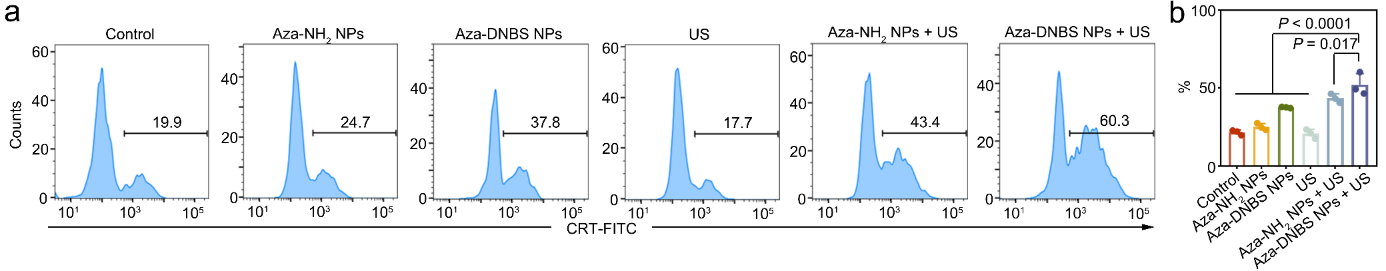
**Figure S9.** (a) Flow cytometry analysis images, and (b) the corresponding statistics illustrating CRT expression on dead cell membranes (PI^+^ cells) after various treatments (n = 3). Statistical significance was calculated by one-way ANOVA.


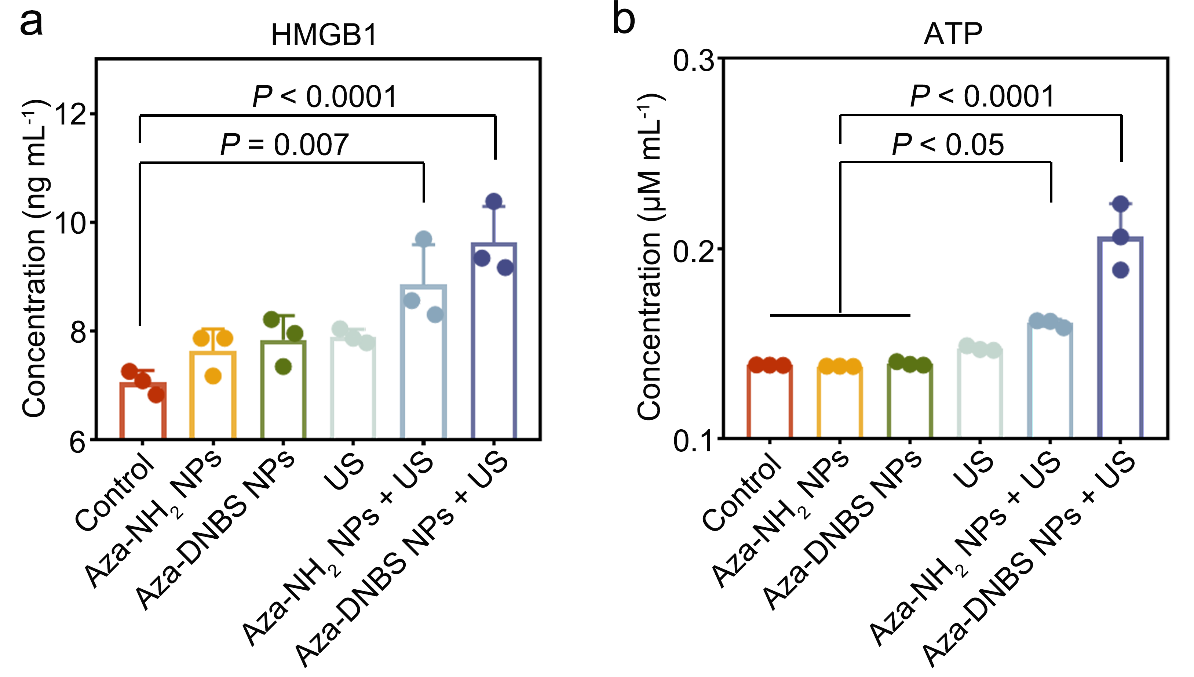


**Figure S10.** The levels of (a) HMGB1 and (b) ATP released in Huh 7 cell culture medium after different treatments (n = 3). Statistical significance was calculated through one-way ANOVA.


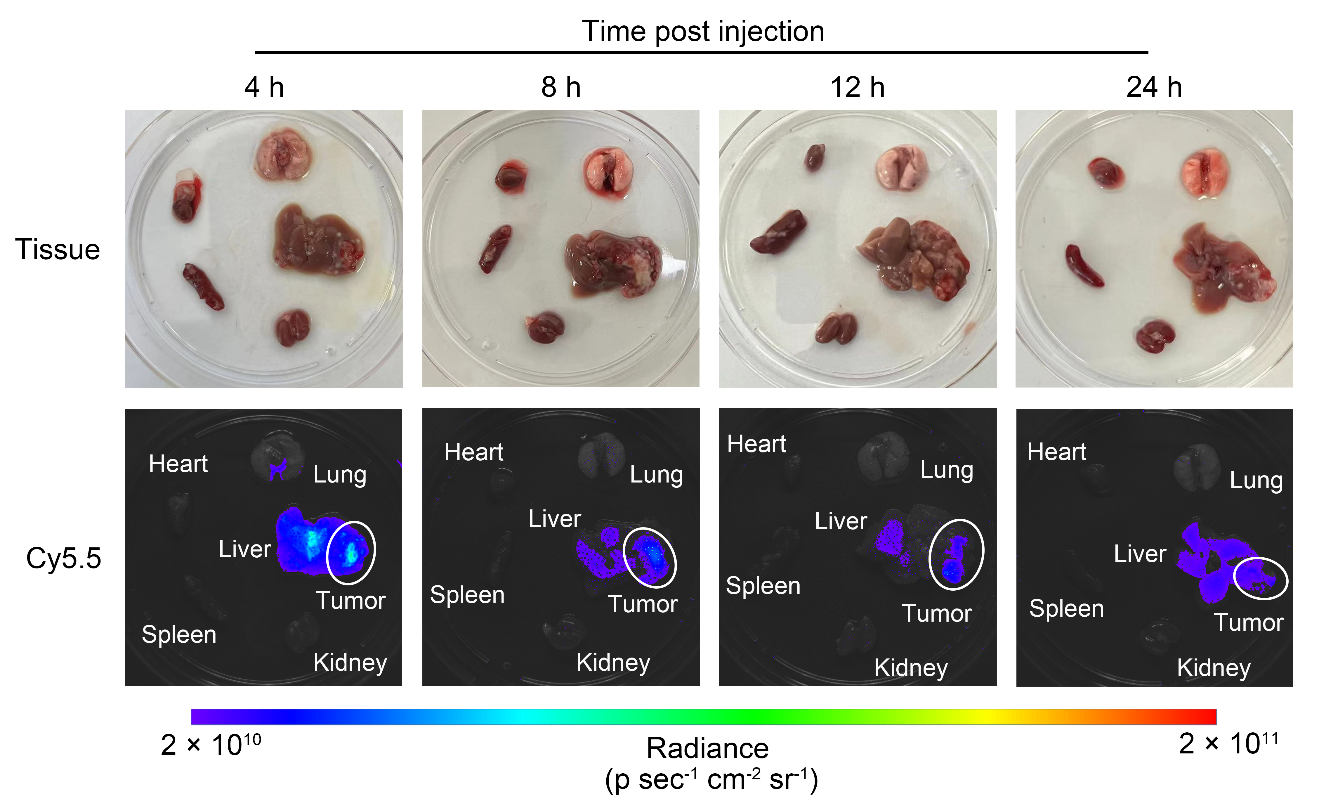


**Figure S11.** Ex vivo fluorescence images showing the distribution of Aza-DNBS NPs in major organs at 4, 8, 12, and 24 h after intravenous injection.


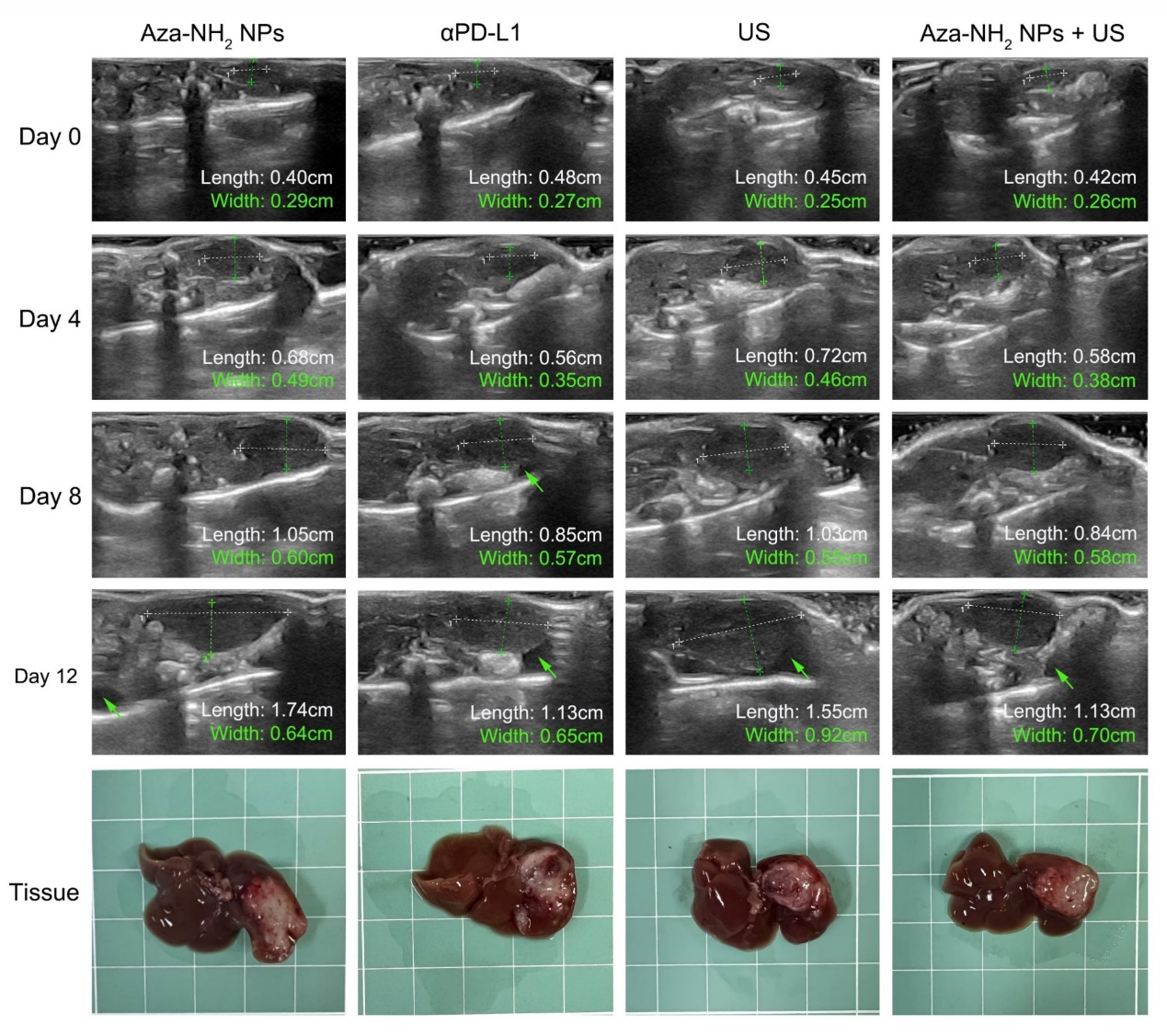


**Figure S12.** High-frequency ultrasound images of H22-Luc orthotopic tumor-bearing mice receiving various treatments on days 0, 4, 8, and 12, accompanied by the corresponding ex vivo tissue images. The green arrows mark the ascites.


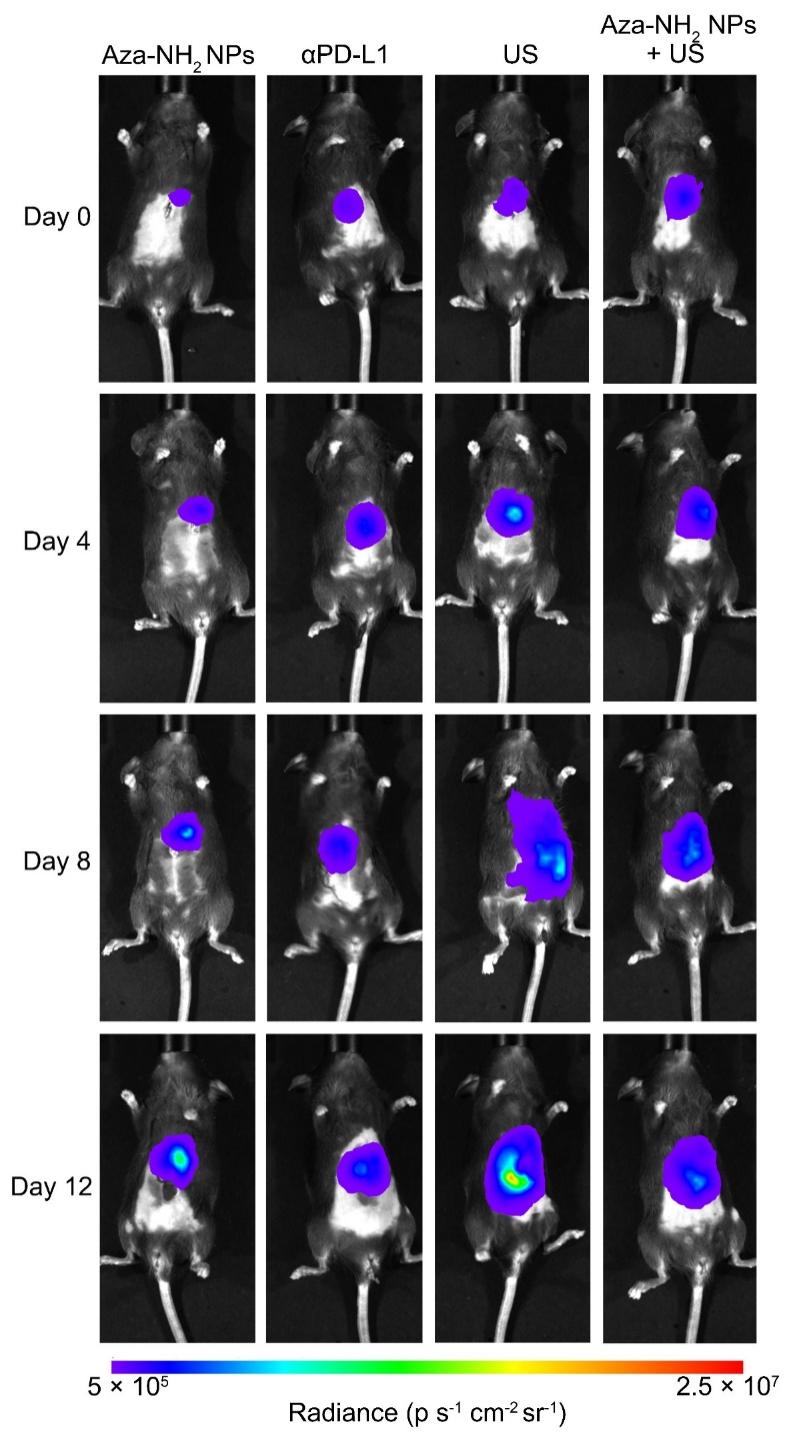


**Figure S13.** Representative IVIS images of the H22-Luc orthotopic tumor-bearing C57BL/6 mice subjected to various treatments on days 0, 4, 8, and 12.


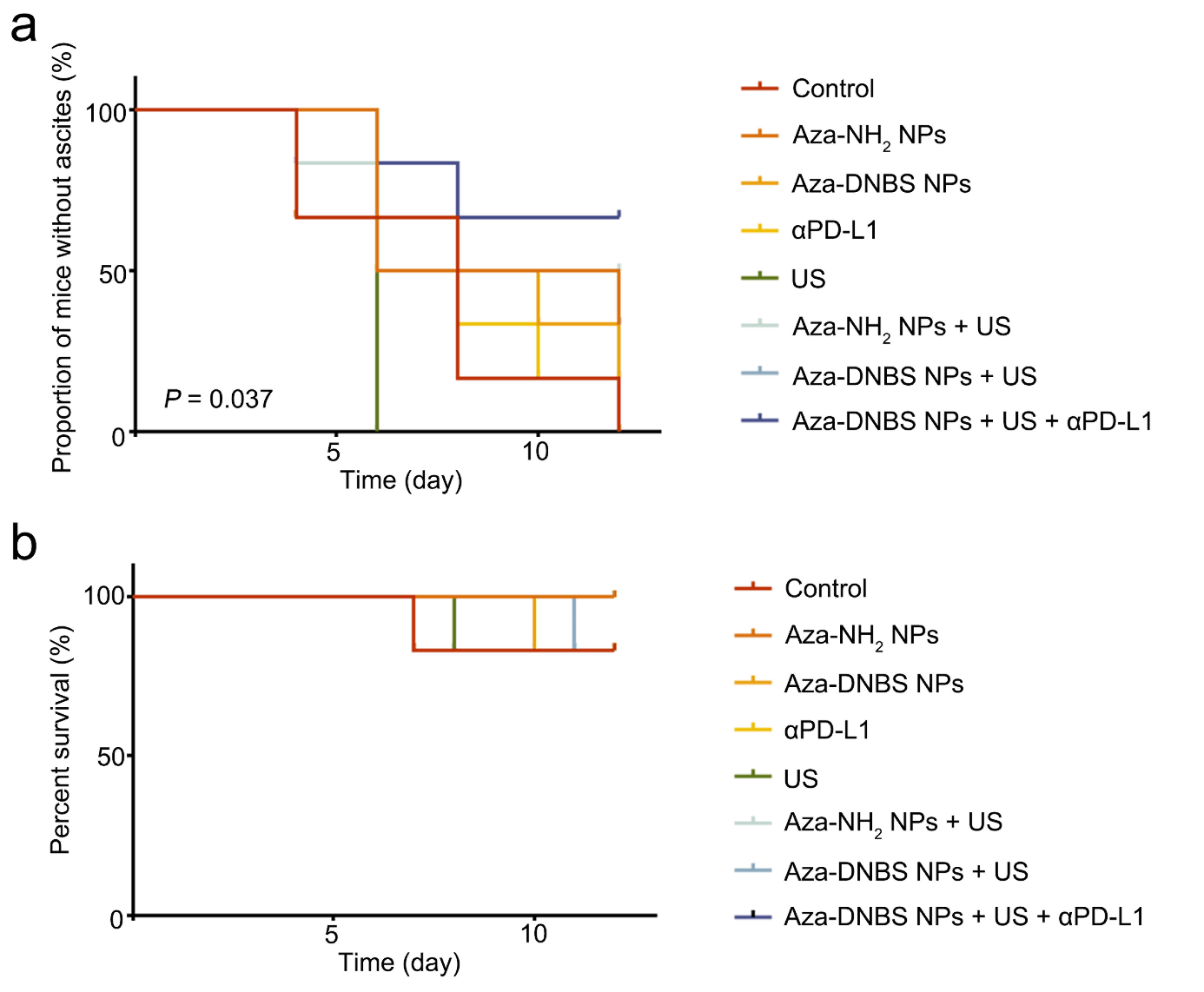


**Figure S14.** (a) Ascites occurrence curves for each group of mice during the treatment period. (b) Survival status of each group of mice during the treatment period.


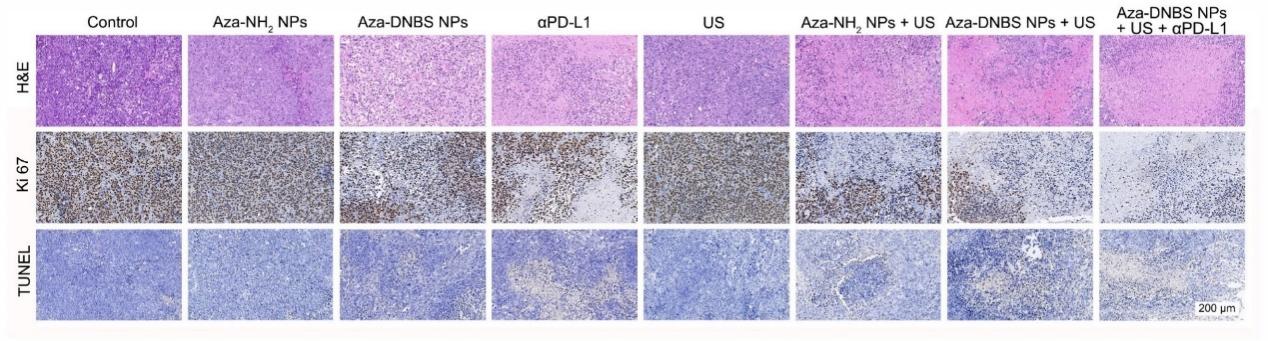


**Figure S15.** H&E, Ki67, and TUNEL staining images of the representative tumor tissues in various treatment groups.


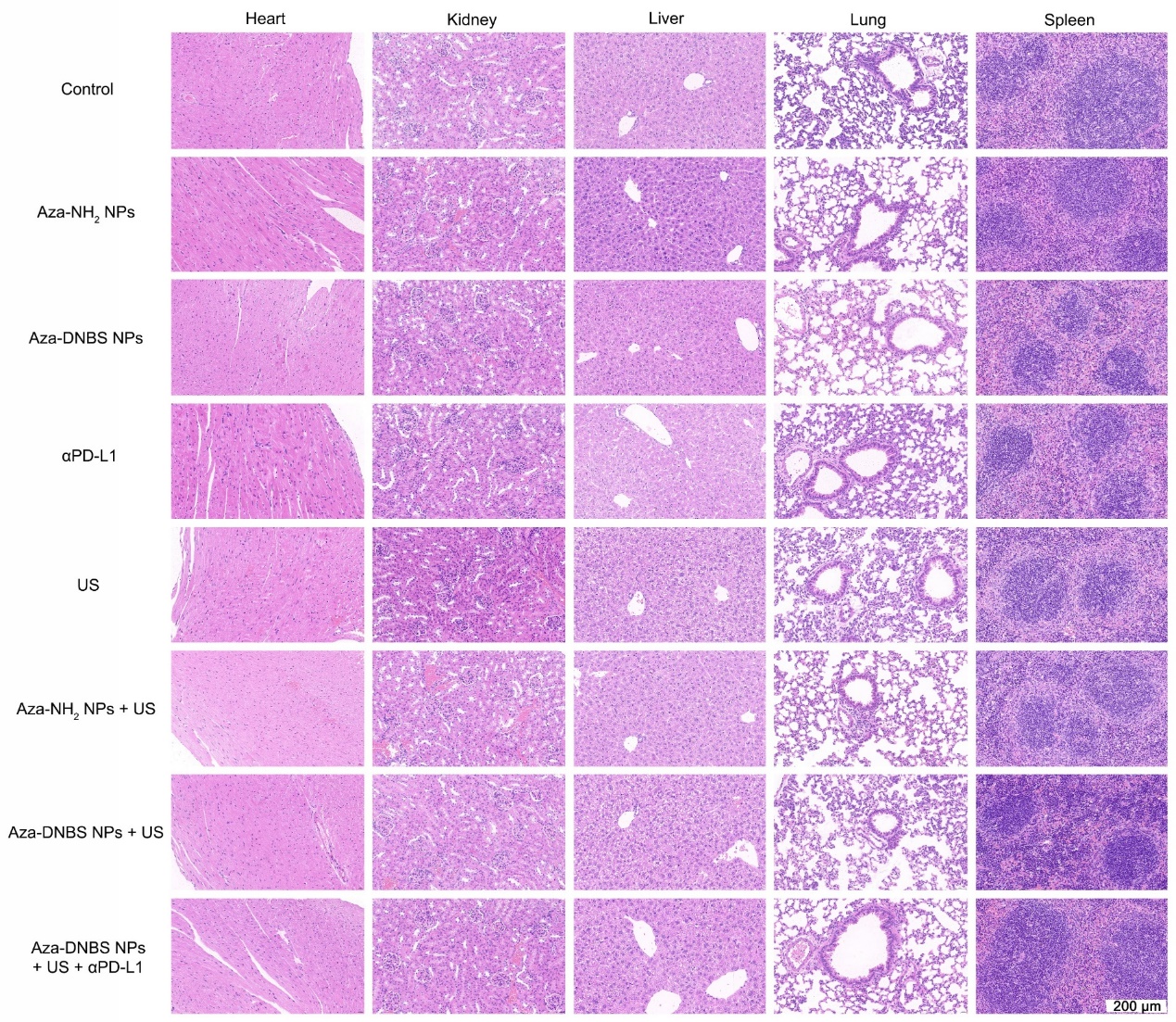


**Figure S16.** Representative H&E staining images of major organs (heart, kidney, liver, lung, and spleen) excised from H22-Luc orthotopic tumor-bearing mice after various treatments.


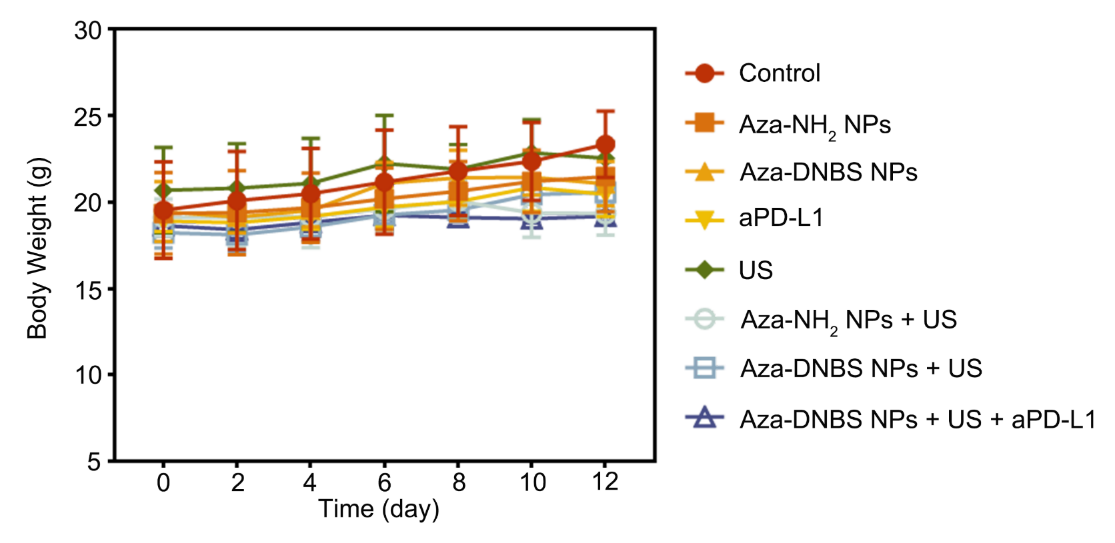


**Figure S17.** Changes in body weights of the H22-Luc orthotopic tumor-bearing mice subjected to diverse treatments.


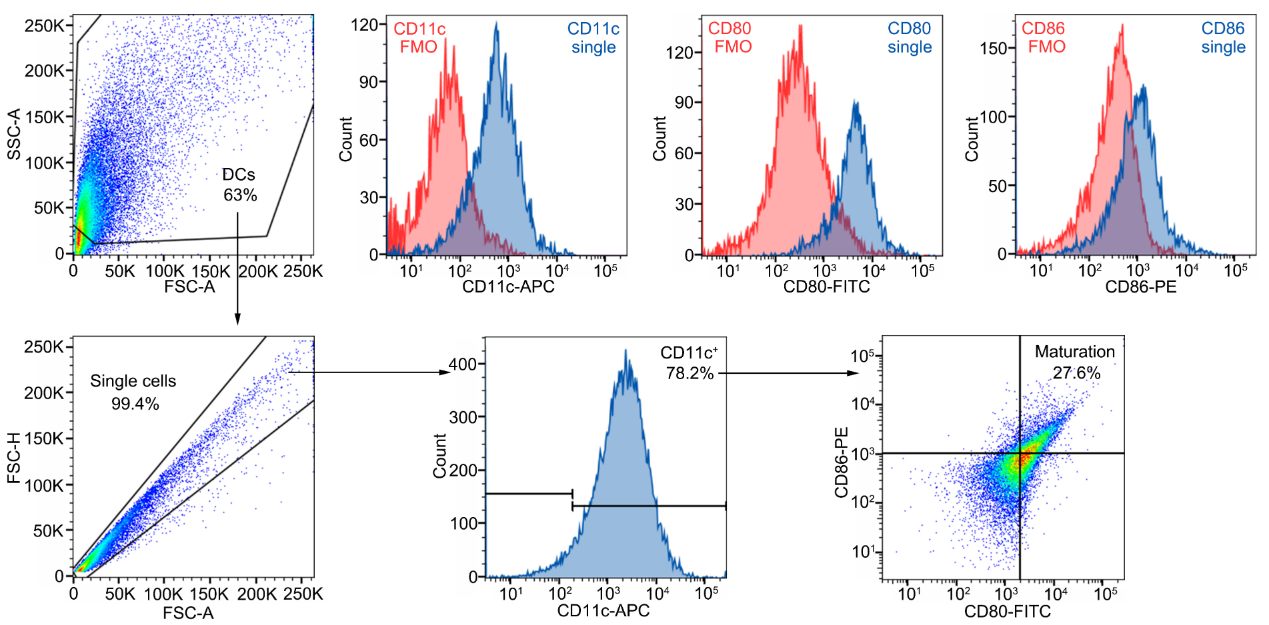


**Figure S18.** Detailed protocol for flow cytometry analysis of dendritic cells (DC) maturation (CD80^+^CD86^+^ proportion in CD11c^+^ cells) in tumor-draining lymph nodes (TDLN). Fluorescence minus one (FMO) controls were performed to determine the cut-off point.


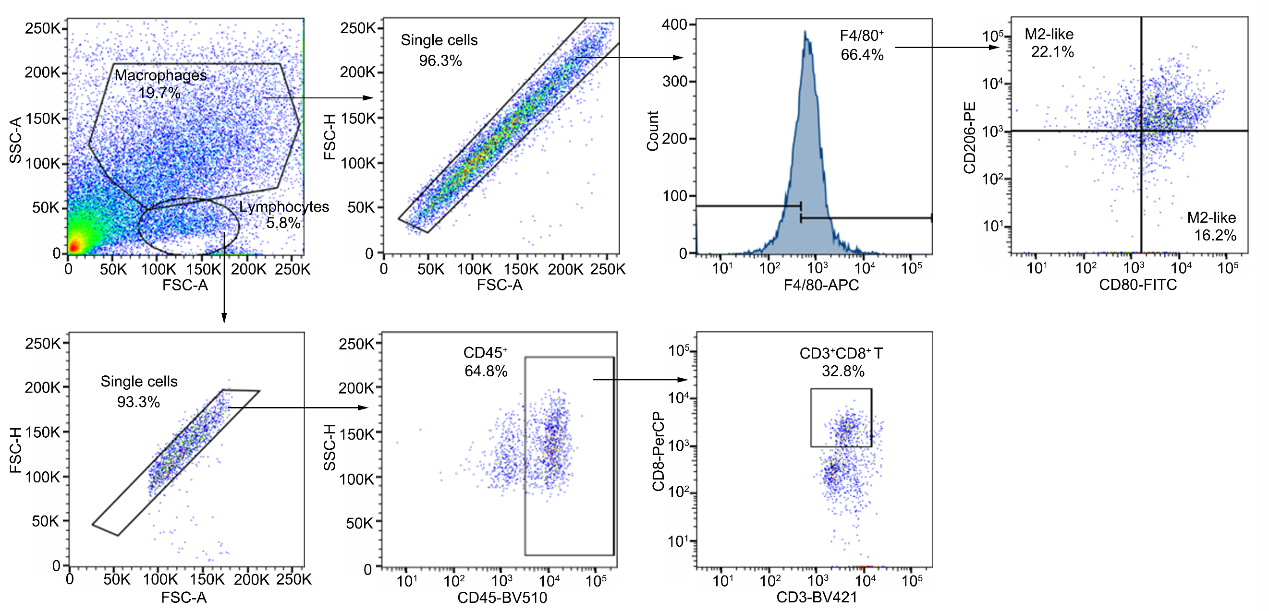


**Figure S19.** Detailed protocol for flow cytometry analysis of the M1-like macrophages (CD80highCD206low in F4/80^+^ cells), M2-like macrophages (CD80lowCD206high in F4/80^+^ cells) and CD8^+^ T cells (CD3^+^CD8^+^ in CD45^+^ cells) infiltrations in the tumors.


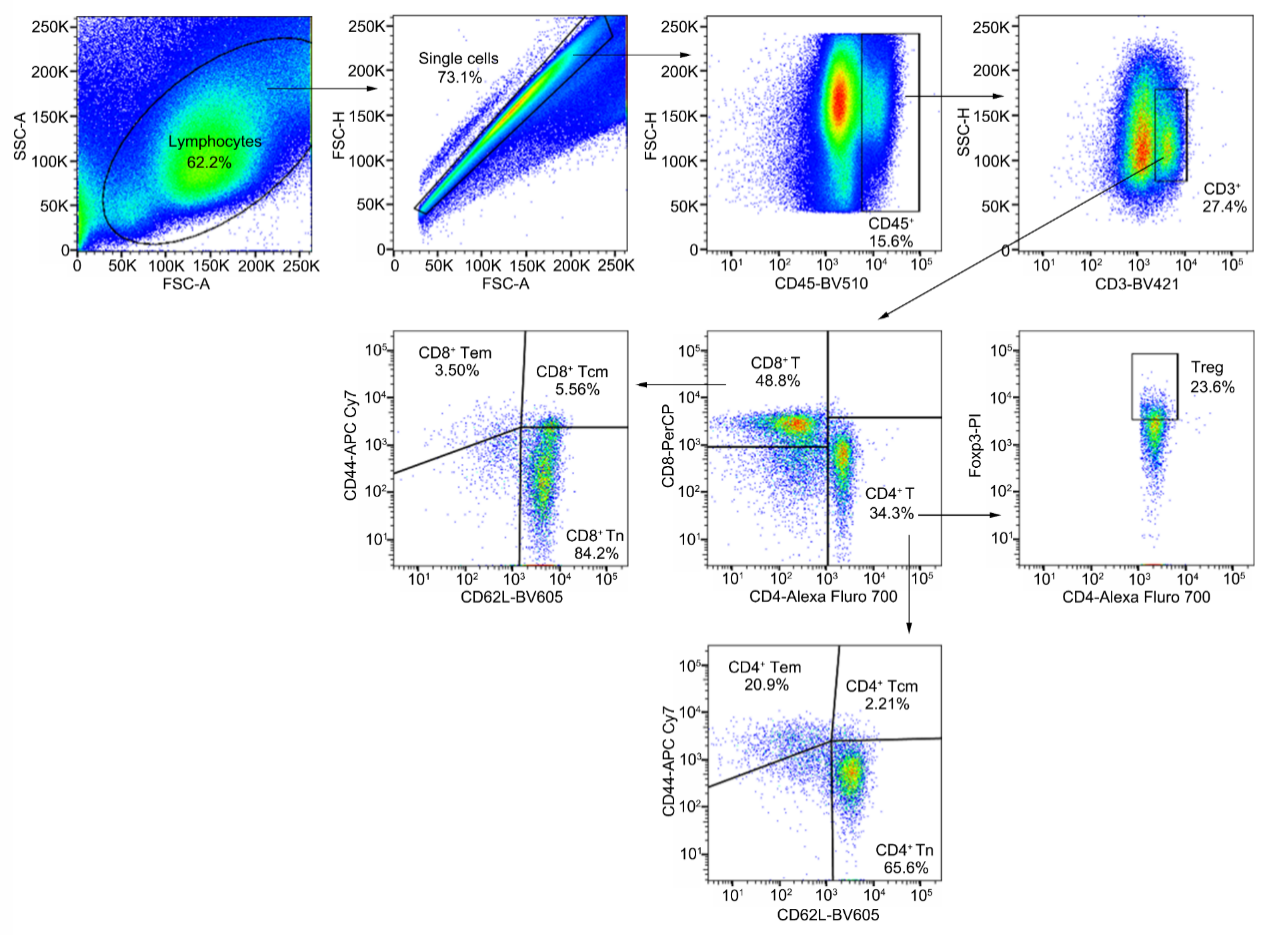


**Figure S20.** Detailed protocol for flow cytometry analysis of the proportions of the CD4^+^ T cells (CD45^+^CD3^+^CD4^+^ cells), CD8^+^ T cells (CD45^+^CD3^+^CD8^+^ cells), Tregs (CD45^+^CD3^+^CD4^+^Foxp3^+^ cells), native T cells (Tn, CD45^+^CD3^+^CD4^+^CD44^low^CD62L^high^ cells), central memory T cells (Tcm, CD45^+^CD3^+^CD4^+^CD44^high^CD62L^high^ cells), and effector memory T cells (Tem, CD45^+^CD3^+^CD4^+^CD44^high^CD62L^low^ cells) in the spleen.


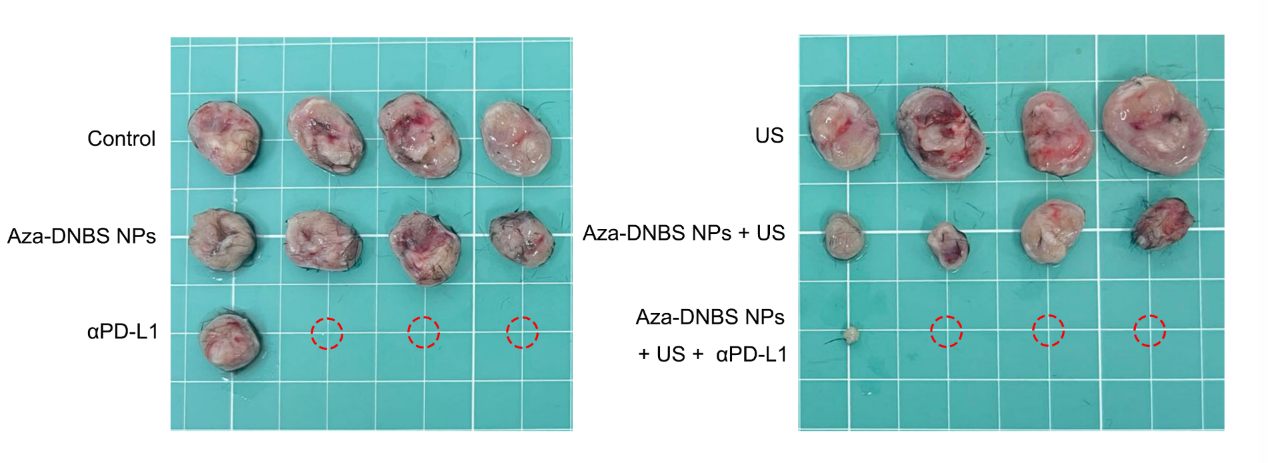


**Figure S21.** Digital photographs of the H22 subcutaneous tumors after diverse treatments.


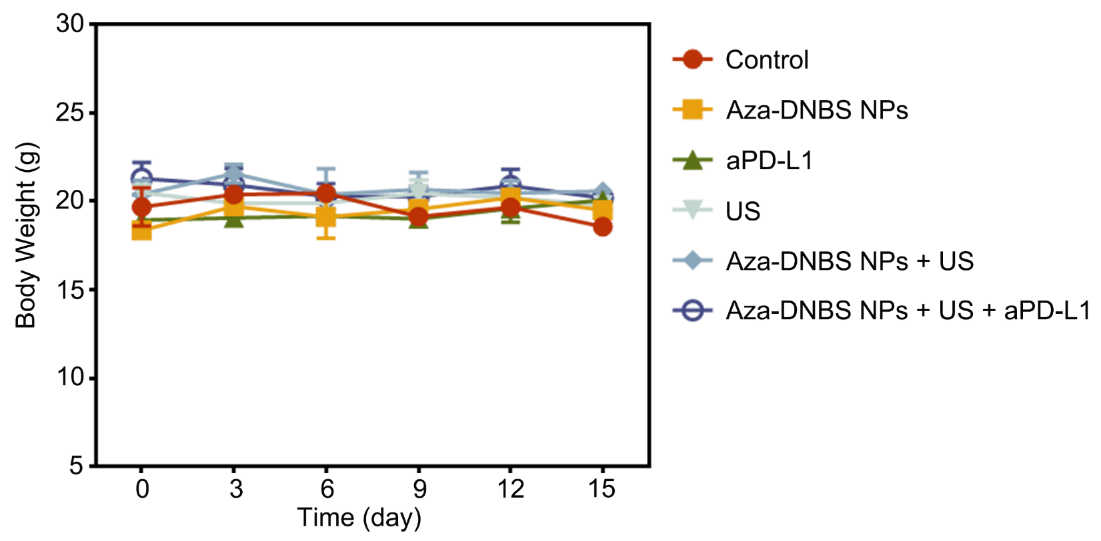


**Figure S22.** Changes in body weights of the H22 subcutaneous tumor-bearing mice throughout the treatment period.


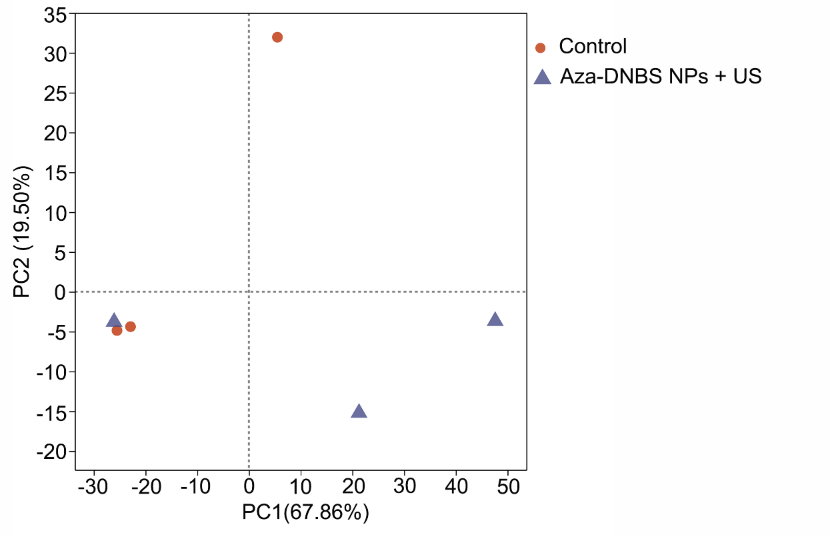


**Figure S23.** Principal component analysis (PCA) scatter plot. Characteristics of control group and Aza-DNBS NPs + US group, according to the gene expression profiles. Each dot indicates a sample.


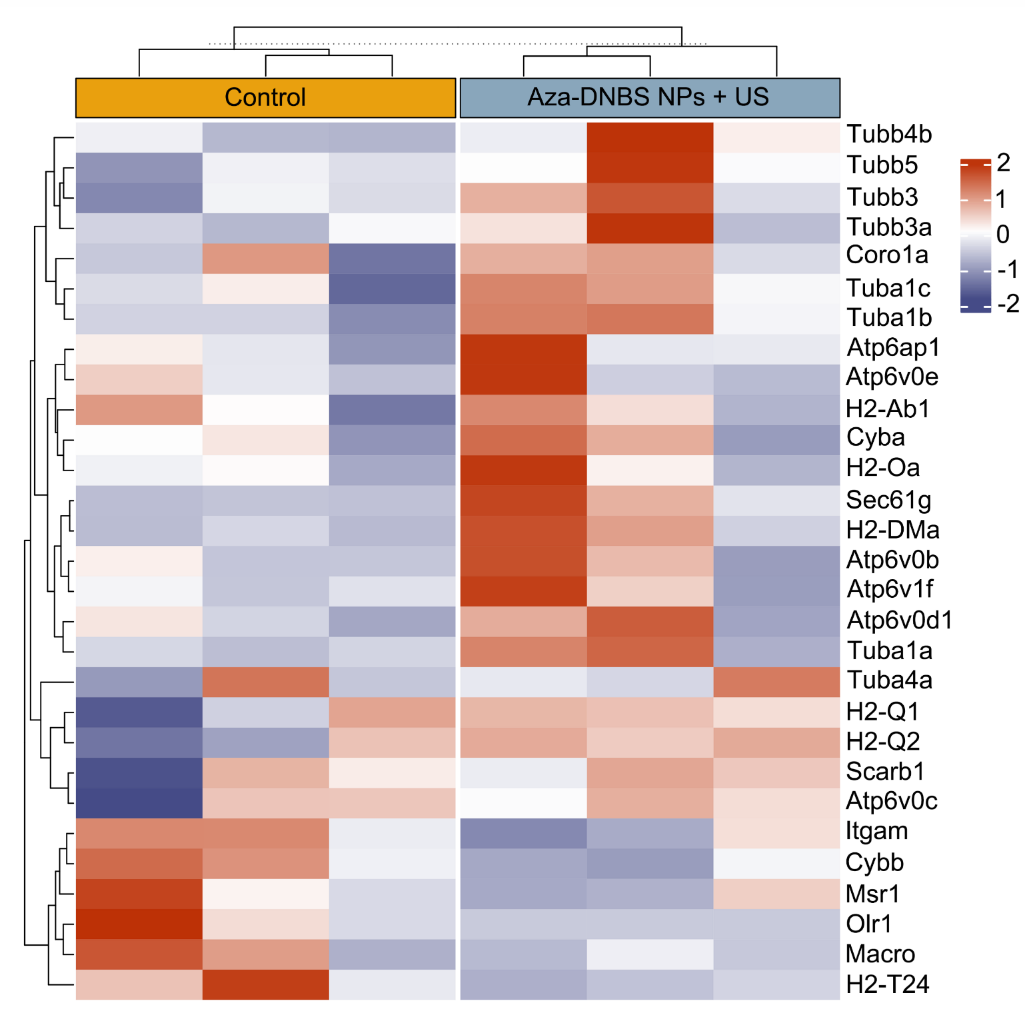


**Figure S24.** The heatmap displaying the changes of DEGs involved in phagosome.


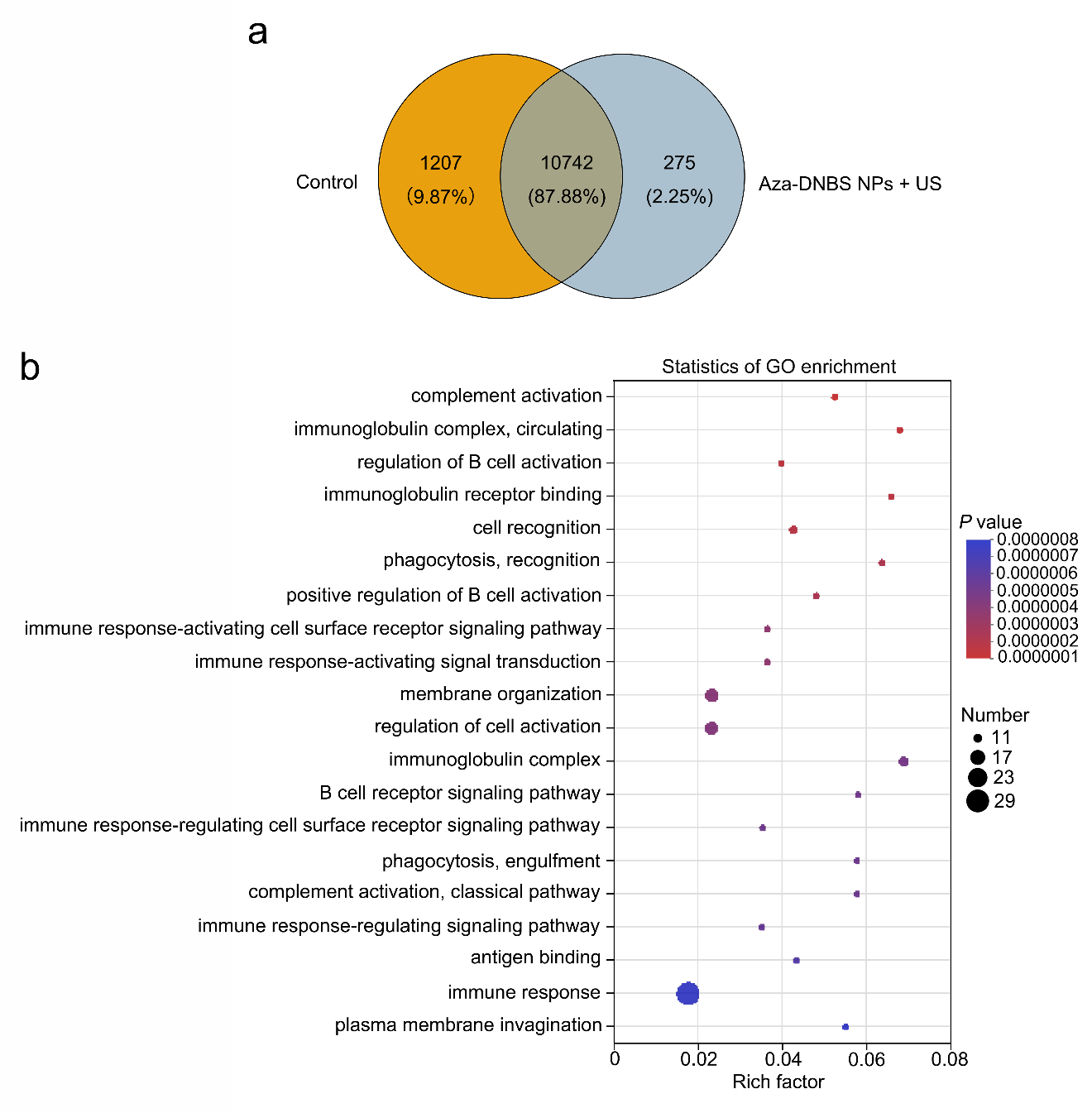


**Figure S25.** (a) Venn diagram indicating 10742 genes are in the intersection of control group and Aza-DNBS NPs + US group, while 275 genes are unique to the Aza-DNBS NPs + US group. (b) GO enrichment analysis of the 275 unique genes.
